# Chronic Mild Traumatic Brain Injury: Aberrant Static and Dynamic Connectomic Features Identified Through Machine Learning Model Fusion

**DOI:** 10.1007/s12021-022-09615-1

**Published:** 2022-12-02

**Authors:** Nicholas J. Simos, Katina Manolitsi, Andrea I. Luppi, Antonios Kagialis, Marios Antonakakis, Michalis Zervakis, Despina Antypa, Eleftherios Kavroulakis, Thomas G. Maris, Antonios Vakis, Emmanuel A. Stamatakis, Efrosini Papadaki

**Affiliations:** 1 Computational Bio-Medicine Laboratory, Institute of Computer Science, Foundation for Research and Technology–Hellas, 70013 Heraklion, Greece; 2 Department of Neurosurgery, School of Medicine & University Hospital of Heraklion, University of Crete, Crete, Greece; 3 Division of Anaesthesia, School of Clinical Medicine, University of Cambridge, Addenbrooke’s Hospital, Hills Rd, CB2 0SP Cambridge, UK; 4 Department of Clinical Neurosciences, School of Clinical Medicine, University of Cambridge, Addenbrooke’s Hospital, Hills Rd, CB2 0SP Cambridge, UK; 5 Department of Psychiatry, School of Medicine & University Hospital of Heraklion, University of Crete, Crete, Greece; 6 Digital Image and Signal Processing Laboratory, School of Electrical and Computer Engineering, Technical University of Crete, Chania, Greece; 7 Department of Radiology, School of Medicine & University Hospital of Heraklion, University of Crete, Crete, Greece

**Keywords:** Traumatic Brain Injury, fMRI, Depression, Verbal Fluency, Functional Connectivity

## Abstract

Traumatic Brain Injury (TBI) is a frequently occurring condition and approximately 90% of TBI cases are classified as mild (mTBI). However, conventional MRI has limited diagnostic and prognostic value, thus warranting the utilization of additional imaging modalities and analysis procedures. The functional connectomic approach using resting-state functional MRI (rs-fMRI) has shown great potential and promising diagnostic capabilities across multiple clinical scenarios, including mTBI. Additionally, there is increasing recognition of a fundamental role of brain dynamics in healthy and pathological cognition. Here, we undertake an in-depth investigation of mTBI-related connectomic disturbances and their emotional and cognitive correlates. We leveraged machine learning and graph theory to combine static and dynamic functional connectivity (FC) with regional entropy values, achieving classification accuracy up to 75% (77, 74 and 76% precision, sensitivity and specificity, respectively). As compared to healthy controls, the mTBI group displayed hypoconnectivity in the temporal poles, which correlated positively with semantic (r = 0.43, p < 0.008) and phonemic verbal fluency (r = 0.46, p < 0.004), while hypoconnectivity in the right dorsal posterior cingulate correlated positively with depression symptom severity (r = 0.54, p < 0.0006). These results highlight the importance of residual FC in these regions for preserved cognitive and emotional function in mTBI. Conversely, hyperconnectivity was observed in the right precentral and supramarginal gyri, which correlated negatively with semantic verbal fluency (r=-0.47, p < 0.003), indicating a potential ineffective compensatory mechanism. These novel results are promising toward understanding the pathophysiology of mTBI and explaining some of its most lingering emotional and cognitive symptoms.

**Supplementary Information:**

The online version contains supplementary material available at 10.1007/s12021-022-09615-1.

## Introduction

Mild Traumatic Brain Injury (mTBI) poses a global health problem of alarming importance, affecting most countries across all continents. It is estimated that half of the world’s population will experience one or more TBIs over their lifetime (Maas et al., 2017). While TBI is a significant cause of death among all age groups, it is the most prominent cause of mortality among young adults (Maas et al., 2017). Although approximately 90% of TBIs are classified as mild (Len and Neary, 2011), no consensus has yet been reached regarding the prevalence and causes of persistent cognitive and emotional sequalae of mild TBI (Iverson et al., 2019; Karr, Areshenkoff, and Garcia-Barrera, 2014; Maas et al., 2017; McInnes et al., 2017; Ruff et al., 2009). Symptoms associated with mTBI, especially cognitive complaints, often resolve within a three-month period, though it is not uncommon for some to become chronic (Levin and Diaz-Arrastia, 2015). It is estimated that as many as 15–30% of patients continue to suffer from mTBI-related symptoms long after initial trauma (Haarbauer-Krupa et al,. 2021) with higher incidence of post-concussive symptoms in clinic-based samples (Vanderploeg, Curtiss, and Belanger, 2005). Such symptoms include headaches, dizziness, vertigo, depression, fatigue, impulsivity, irritability, as well as a wide range, of possibly heterogenous cognitive deficits (Bell et al., 1999; Konrad et al., 2011; Vanderploeg, Curtiss, and Belanger, 2005). Emotional difficulties, in particular, which may in part be attributed to psychological factors such as poor coping styles (Marsh and Smith, 1995), have also been linked to aberrant connectomic features (Moreno-López et al., 2016).

Conventional anatomical MRI is sensitive at detecting structural traumatic lesions, although the extent of subtle structural impairment is typically underestimated by MRI, especially in mTBI. Functional imaging approaches are, in principle, more sensitive to disturbances in brain function at both the regional and network level toward accounting for physical, emotional, and cognitive symptoms following TBI and aid prognosis of behavioral and cognitive patient outcomes. However, the potential contribution of functional imaging is thwarted by considerable patient heterogeneity in (i) trauma location, type and force, (ii) post-injury symptom profiles and, (iii) the broad range and diversity of rehabilitation practices across hospitals, rehabilitation centers, and geographical regions (Moller, Lexell, and Ramsay, 2021; Rytter et al., 2021). These considerations render the need for novel approaches that preserve individual variability in the indices derived by functional imaging.

The prevalence of studies exploring rs-fMRI-derived large-scale functional networks have facilitated our understanding of the typical and aberrant functional connectome (Luppi et al., 2019; Nathan et al., 2014; Rolls, Cheng, and Feng, 2021; Zhang et al., 2016). The body of functional neuroimaging research on the consequences of mTBI is steadily growing (Sharp, Scott, and Leech, 2014), although the majority of resting-state functional connectome studies focus on the acute and sub-acute phases. However, there is significant variability in the methodological choices employed for the study of regional functional roles and relationships, leading to results than cannot be compared or combined for straightforward interpretations in terms of regional effects of mTBI pathology and symptomatology. Seed-based FC approaches, which compute activity relationships between a brain region and the rest of the brain, have revealed disturbances in the sensorimotor, visual, salience, ventral and dorsal attention, and frontoparietal networks, as well as in several regions of the Default Mode Network (DMN) (Champagne et al., 2020; Madhavan et al., 2019; Sours et al., 2015). Nathan et al. (2014) utilized a more data-driven approach based on Independent Component Analysis (ICA), examining the DMN and sensorimotor networks. Their findings included decreased DMN connectedness in the left inferior temporal and right precentral gyri as well as left caudate and inferior parietal lobule. Additionally, the same team reported increased DMN connectedness in the bilateral posterior cingulate and temporal regions. Using a similar methodology, semi-acute mTBI patients (N = 50) were effectively distinguished (84% AUC) from healthy individuals (N = 50) (Vergara et al., 2016). Elevated connectivity was observed between the inferior parietal cortex and posterior precuneus (of the DMN) as well as between the cerebellum and sensorimotor networks (Vergara et al., 2016). Notably, a re-analysis of the latter dataset using a seed-based approach found decreased connectivity in mTBI patients within the DMN and increased connectivity between the DMN and lateral prefrontal cortex (Mayer et al., 2011).

Recently, there is a growing consensus that functional brain connectivity is not static, but rather it varies from moment to moment, exhibiting dynamics that have been shown to play important roles in both healthy and pathological cognition and even consciousness (Luppi et al., 2019; Luppi et al., 2021; Lurie et al., 2020; Rolls, Cheng, and Feng, 2021; Shine et al., 2016; Sun et al., 2019; Zhang et al., 2016). Dynamic functional connectivity (DFC) principles have been applied to the study of mTBI: using dynamic states of FC as inputs for a machine learning classifier, Vergara et al. (2018), were able to effectively distinguish between semi-acute stage mTBI patients and healthy controls at an impressive accuracy of 87–92%. Significant findings were reported in cerebellum and sensorimotor areas (Vergara et al., 2018) (but see Mayer et al., 2014, for conflicting results). However, it is clinically imperative to assess FC changes that persist into the chronic phase, as these are more likely to be associated with the long-term functional consequences of mTBI. To our knowledge, there is no work on chronic mTBI FC disturbances using dynamic, or dynamic combined with static, functional connectivity.

The present study integrates multiple functional metrics, including static and dynamic FC measures and regional activation complexity over time, to assess persisting connectomic disturbances following mTBI through the study of individual patient connectomes. The second aim of the present analyses was to assess the functional significance of aberrant connectomic patterns to account for the lingering effects of mild head trauma on emotional status (i.e., anxiety and depression symptoms) and cognitive function (performance on standardized tests of episodic memory, executive function, and language). We employ a novel machine learning approach followed by statistical thresholding and correction to ensure robust and conservative data-driven cross-validation.

## Materials and methods

### Participants

The dataset of the present study comprises rs-fMRI scans from 37 patients with mTBI obtained in the chronic phase, as well as healthy controls (HC) (n = 39). Initially, 46 patients meeting inclusion criteria were identified through the registry of the Neurosurgery Clinic, Heraklion University Hospital and invited to return for follow-up MRI and neuropsychological assessment. Inclusion criteria were the following: (a) age at the time of injury 19–65 years, (b) non-penetrating injury that did not require neurosurgical intervention, (c) mild injury severity as indicated by Glasgow Coma Scale (GCS) score ≥ 13 upon admission (Russell and Smith, 1961; Teasdale and Jennett, 1974), and (d) time elapsed since brain injury ≥ 6 months. Exclusion criteria were: (i) History of neurological or psychiatric disease prior to injury, current history of substance abuse, or currently receiving psychoactive medications other than anticonvulsants, (ii) Posttraumatic multifocal or unifocal extensive lesions (i.e., gliotic areas due to contusions > 3 cm or multiple (> 3) chronic hemorrhagic foci resulting from diffuse axonal injuries (DAIs)) at the time of inclusion. Nine patients did not meet the inclusion criteria and were not included in the analyses. Detailed social/psychiatric history at the time of testing revealed that none of the patients were involved in litigation concerning their injury or indicated that the results of the study could be used for seeking compensation since this practice is not customary in Greece. Moreover, they had not received systematic psychiatric or psychological interventions post-injury. All participants in the HC group underwent a structured interview to record basic demographic information and ensure that they did not meet the exclusionary criteria (history of neurological [including TBI] or psychiatric disease, current history of substance abuse, or currently receiving psychoactive medications, without undergoing comprehensive neuropsychological testing.

Time post injury at the time of the MRI and neuropsychological evaluation averaged 26.3 months (SD = 15.5). The two groups (patients and HCs) were closely matched on age (mTBI mean = 40.33, SD = 17.4 years, HC mean = 41.73, SD = 15.6 years), although the former group included a higher percentage of men (84% vs. 72%, p = 0.2), and had achieved higher formal education (mTBI mean = 11.72, SD = 3.8 years, HC mean = 13.9, SD = 4.0 years, p = 0.01). The study was approved by the University Hospital Ethics Review Board, details of the procedure was explained to all participants, who provided written informed consent.

### Neuropsychological Assessment

The cognitive and emotional status of all mTBI patients was assessed on the same day as the MRI session, using a battery of standardized tests, available in Greek. Tests covered a wide range of cognitive domains in view of the reported heterogeneity of patient neurocognitive profiles, especially in the chronic phase (see meta-analyses by McInnes et al., 2017 and Karr, Areshenkoff, and Garcia-Barrera, 2014). The following tests were administered: Memory for Digits Forward and Reverse subtests of the Greek Memory Scale (Constantinidou et al., 2014; Simos et al., 2011) to assess short-term and working verbal memory; The Passage Memory subscale of the Greek Memory Scale and delayed reproduction of the modified Taylor Complex Figure test (TCF) (Hubley and Tremblay, 2002) to assess secondary episodic memory. The Trail Making Test (TMT) Part A and B were used to assess visuomotor coordination speed and mental flexibility (Zalonis et al., 2008). Semantic (SVFT) and Phonetic (PhVFT) subtests of the verbal fluency test were employed for assessment of strategic rule-based access to stored lexical representations (Kosmidis et al., 2004). The Matrices subtest of the Wechsler Adult Intelligence Scale (WAIS-IV) indicated problem solving ability (Wechsler, 2008). All aforementioned neuropsychological measures were converted to z scores based on Greek population norms (adjusted for age and education). Furthermore, the Greek adaptations of the Center for Epidemiology Studies Depression Scale (CESD) (Fountoulakis et al., 2001) and Spielberger Trait Anxiety Inventory (STAI-B) (Fountoulakis et al., 2006) were used for assessment of depression and anxiety symptoms. Demographic, clinical, and neuropsychological data for the mTBI group are presented in Table 1.

**Table 1:** **Clinical, demographic, and neuropsychological information of** mTBI patients

	N(%) / Mean ± SD	Range
Age (years)	40.3 ± 17.4	19–65
Education (years)	11.72 ± 3.8	6 to 22
Gender: Men (n/%)	31 (84%)	-
Trauma type: MVA (n/%) Fall (n/%)	20 (54.1%) 17 (45.9%)	- -
Months post injury	18.7 ± 11.7	6 to 60
GCS^1^	14.55 ± 0.9	13 to 15
CESD^1^	12.30 ± 8.8	0 to 35
CESD > 22 (n/%)	8 (21.6%)	-
STAI-B^1^	46.11 ± 10.5	32 to 75
STAI-B > 49 (n/%)	18 (48.6%)	-
Digits Forward^2^	-0.67 ± 0.8†	-2.6 to 1.2
Digits Reverse^2^	-0.61 ± 0.7†	-2.3 to 0.7
PM-Immediate^2^	-1.10 ± 1.1†	-3.7 to 1.0
PM-Delayed^2^	-1.17 ± 1.0†	-3.4 to 0.6
PM-Retention^2^	-0.25 ± 1.1	-2.5 to 2.1
PM-Recognition^2^	-0.31 ± 2.0	-2.4 to 1.2
TCF-Copy^2^	0.38 ± 0.8	-1.9 to 1.3
TCF-Memory^2^	-0.12 ± 1.1	-2.1 to 2.6
TMT-A^2^	0.60 ± 0.9	-2.1 to 2.0
TMT-B^2^	0.38 ± 0.9	-3.0 to 2.4
SVFT^2^	-0.55 ± 0.9†	-2.5 to 1.1
PhVFT^2^	-1.14 ± 0.7†	-3.0 to 0.4
WAIS-IV Matrices^2^	-1.01 ± 1.1†	-2.8 to 2.0

Abbreviations; GCS: Glasgow Coma Scale, CESD: Center for Epidemiological Studies Depression scale, STAI-A: State-Trait Anxiety Inventory Form Y (Part B-Trait Anxiety), MVA: Motor Vehicle Accident, TCF: Taylor Complex Figure Test. PM: Passage Memory. TMT: Trail Making Test (Part A and Part B), SVFT: Semantic Verbal Fluency Test, PhVFT: Phonemic Verbal Fluency Test, WAIS-IV: Wechsler Adult Intelligence Scales.

†Significant difference from age- and education-adjusted population mean (p ≤ 0.001); ^1^Raw scores, ^2^Age- and education-adjusted z scores.

### Imaging Data Acquisition

All participants underwent brain MRI scans at the MRI Unit, University Hospital of Heraklion using identical scanning parameters. MRI scans were acquired on a clinical, upgraded 1.5T whole-body superconducting imaging system (Vision/Sonata, Siemens/Erlangen), equipped with high performance gradients (Gradient strength: 40 mT/m, Slew rate: 200 mT/m/ms), and a two-element circularly polarized head array coil (minimum voxel dimensions: 70 μm × 70 μm × 300 μm). The main imaging protocol consisted of a 3D T1-w MPRAGE (TR/TE: 1570/1.73 ms, 1 mm/1 NEX/160 axial sections), a T2wTSE (TR/TE: 5000/98 ms, 4 mm axial sections), and a Turbo FLAIR (TR/TE/TI: 9000/120/2320 ms, 4 mm axial sections) sequence. Axial sections were acquired parallel to the plane passing through the anterior and posterior commissures (AC–PC line). Structural MR images were interpreted by a senior neuroradiologist (Dr E. Papadaki, MD, PhD) with 20 years of experience. Rs-fMRI sequences were acquired using a T2*-weighted, fat-saturated 2D-FID-EPI sequence with repetition time (TR) 2320 ms, echo time (TE) 50 ms, field of view (FOV) 192 × 192 × 108 (x, y, z). Whole brain 3D images consisted of 36 transverse slices with 3.0-mm slice thickness and no interslice gap. Voxel BOLD time series consisted of 150 dynamic volumes, while the voxel size was 3 × 3 × 3 mm. Acquisition duration was ~ 6 min.

### Data Preparation: Preprocessing, Denoising and Parcellation

Initial data preparation steps are in line with several rs-fMRI studies (Luppi et al., 2019; Luppi, Carhart-Harris et al., 2021), including previous work of our team on data from the same MRI system (Antypa et al., 2021; Kavroulakis et al., 2021; Pentari et al., 2022; Simos et al., 2019; Simos et al., 2020). Firstly, the first three volumes were discarded to allow for magnetization effects to stabilize. Slice-timing correction (corrected for *Siemens*-interleaved slice acquisition), re-alignment, and co-registration/normalization to standard MNI space were subsequently performed. Lastly, spatial smoothing with a 6 mm FWHM (Full Width at Half Maximum) gaussian kernel was applied to improve SNR. These steps were carried out in SPM12 (www.fil.ion.ucl.ac.uk/spm-statistical-parametric-mapping/) implemented in MATLAB version 9.8 (R2020a).

Next, mean white matter and cerebrospinal fluid (CSF) signals (first five principal components and their first order derivatives) were regressed out of the voxel time series, using CompCor (Behzadi et al., 2007) included in CONN (Whitfield-Gabrieli and Nieto-Castanon, 2012). Then, voxel timeseries were detrended and bandpass filtered to 0.008–0.09 Hz to eliminate low frequency drift and high frequency noise.

Brain parcellation was implemented into 200 cortical (Schaefer et al., 2018) and 32 subcortical regions (Tian et al., 2020). This resolution and combination of functional atlases was shown to produce the most representative and reproducible large-scale functional networks (Luppi and Stamatakis, 2020; Luppi, et al., 2021). On average, cortical and subcortical regions comprised 660 ± 264 and 247 ± 143 voxels, respectively. BOLD timeseries of voxels belonging to each region were averaged to obtain representative, regional timecources, which were then used to compute functional indices.

### Functional Connectivity Analysis

#### Static Functional Connectivity (SFC)

Pairwise ROI-ROI FC was computed between all pairs of regions using the Pearson correlation coefficient (Prsn) or Mutual Information (MI) producing two Static Functional Connectivity Graphs (SFCGs) per subject, one for each connectivity estimation method, each of size 232 × 232. Two complementary connectivity measures were utilized to encapsulate synergistic functionality apparent through both linear and non-linear patterns of coactivation.

#### Dynamic Functional Connectivity (DFC)

Dynamic FC metrics were computed to assess time-varying features of regional coactivation over shorter timescales (compared to SFC that refers to the entire scan). A tapered maximum overlapping sliding window approach was utilized for DFC calculation (Supplementary Fig. 5) (Allen et al., 2012). A window length of 23 TRs (53 s) was used in line with the proposed range of 30–60s (Bijsterbosch et al., 2017) overlapping on a single TR. Tapered windows were used, achieved by convolving them with a Gaussian kernel of σ = 3TRs. In this manner, 125 windows were created, of 23 TRs length each. Pairwise ROI-ROI FC was estimated within each temporal window using the Prsn, resulting in 125 time-resolved networks or DFCGs (of size 232 × 232 each). The MI metric was not utilized for DFC in view of theoretical concerns that its computation is not suitable for shorter length timeseries, and results can be potentially unreliable.

To uncover the dynamics of predominant recurring connectomic patterns, a brain state identification process based on *”cartographic profiling”* was utilized (Fukushima, Betzel, He, van den Heuvel et al., 2018; Luppi et al., 2019; Luppi, Carhart-Harris et al., 2021; Shine et al., 2016). As depicted in Supplementary Fig. 6, the goal of this approach is classifying time-resolved networks as predominately integrated or segregated based on the most prominent functional network tendencies. Integrated networks tend to be globally well-connected and efficient, while segregated networks consist of several separated communities of strongly connected nodes and few connections between communities.

**Fig. 1:**
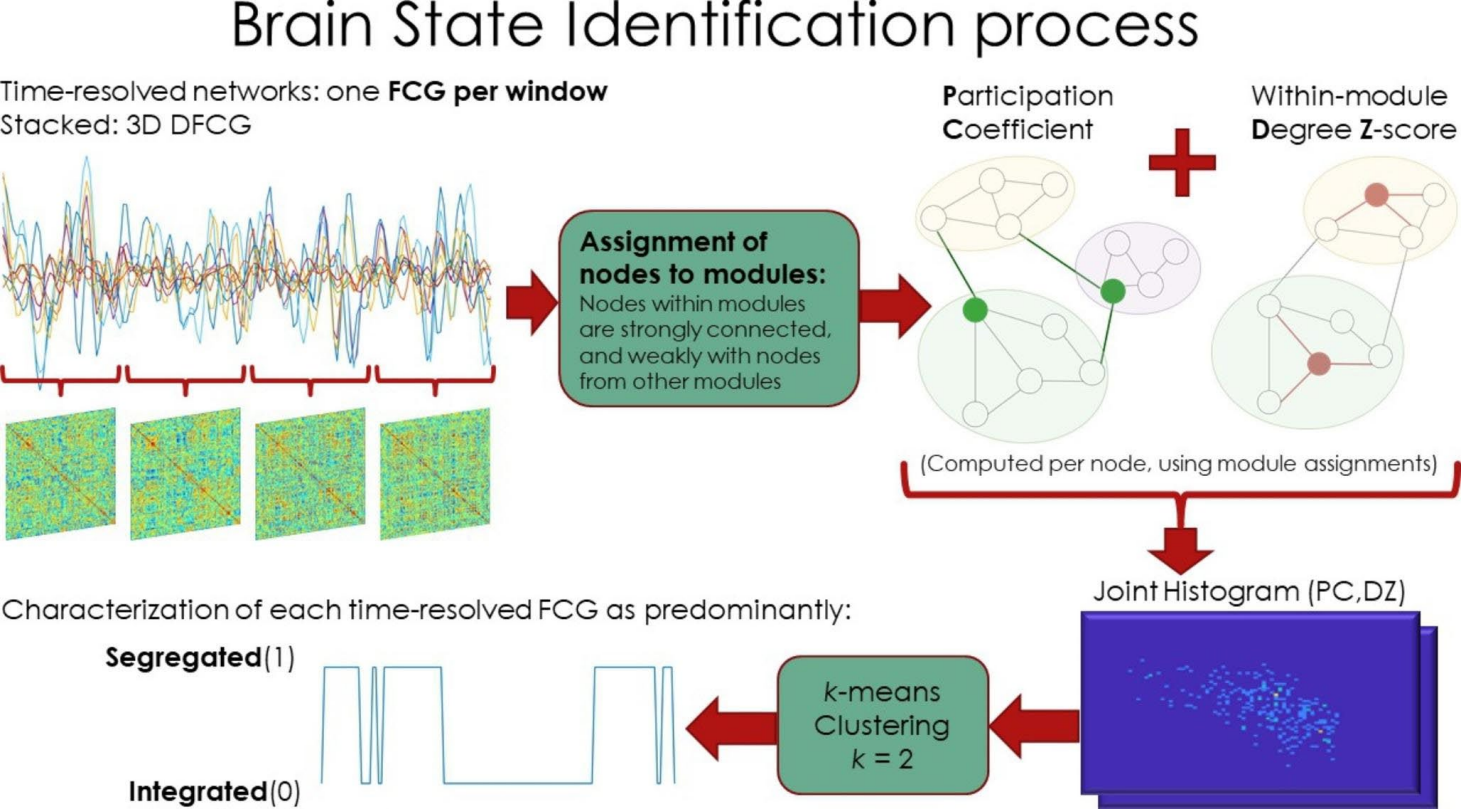
Pipeline used in the identification of Integrated and Segregated dynamic states

The brain state identification process is depicted in Fig. 1. Firstly, modules are identified in each time-resolved subject-level network. Functional modules or communities are groups of nodes strongly connected to each other and less so (or anticorrelated) with nodes of other modules. The Louvain algorithm was used for community detection, which operates in a greedy fashion by repeatedly assigning nodes to modules until the modularity value of the network is maximized, denoting an ”optimal” split of the network. The modularity value quantifies the overall decomposability of the network. Two complementary network measures are utilized to characterize each node’s inter- and intra- modular connectivity, participation coefficient and within-module degree z-score respectively. Collaboratively, the two metrics are theoretically able to encapsulate the network’s tendencies towards global integration or segregation. Their joint histogram or *”cartographic profile”* (Shine et al., 2016) is used to cluster the 125 DFCGs (per participant) into *k* = 2 clusters using *k*-means clustering (see also Supplementary Methods). Finally, a representative network for each state is computed as the edgewise median of the DFCGs assigned to each state. Only positive FCG values were retained from this step onward.

#### Graph Reduction: Optimal Network Structure

Fully-weighted, fully-connected, subject-specific FCGs are considered sub-optimal and potentially unable to reveal the true characteristics of the underlying network. Furthermore, the very large number of initial connections is biologically implausible and may include spurious connections driven predominately by noise. With this motivation, functional networks were reduced to derive the “true” underlying network structure, while at the same time preserving systematic variability of the final networks among participants. OMST (Dimitriadis, Antonakakis et al., 2017; Dimitriadis et al., 2017) was employed for this task. OMST is a technique capable of generating highly reproducible and overall representative FC networks (Luppi and Stamatakis, 2020; Luppi et al., 2021; for additional details see Supplementary Methods section).

#### Functional Network Measures

A wide range of global and nodal graph measures were utilized to capture and quantify various functional and topological network characteristics and reveal mTBI-related functional disturbances. This specialized feature extraction step enables the comparison of networks exhibiting varying density and topology. Nodal (region-specific) graph measures reflect increased communication efficiency among the nodes comprising the immediate local community (local efficiency), and the node’s ability to act as a go-between, facilitating the stability of a broader network (degree, betweenness or eigenvector centrality). Conversely, global, network-wide measures, reveal the integrative tendencies and performance of the entire network (global efficiency), as well as indicate the presence of complex topologies, and the balance between integration-segregation and information capacity (Barttfelda et al., 2015). The latter, is based on the notion of small world networks (Bassett and Bullmore, 2017; Rubinov and Sporns, 2010; Watts and Strogatz, 1998) and can be measured by the metric of small world propensity (Muldoon, Bridgeford, and Bassett, 2016). Thus, both local and global measures are suitable to identify regions displaying either hypo- or hyper-connectivity associated with mTBI. Graph metrics were combined/concatenated to form a single feature vector (of size 2(global) + 4(nodal) x 232(nodes) = 930 features per subject) for each of the 4 functional networks (derived from Prsn and MI [static connectivity measures] and from the integrated and segregated networks [dynamic connectivity measures]).

In addition, sample entropy was calculated for each regional time series to quantify the unpredictability or irregularity of regional activation over time and to aid interpretation of potential group differences in regional FC. Sample entropy has been shown to be a robust and sensitive signal complexity measure in previous fMRI experiments, with lower entropy values found during task performance and anesthesia. In the former case, signal amplitude and variability as well as regularity and temporal predictability are increased; in the latter case, signal amplitude and variability may be reduced, although regularity is also high (Wang et al., 2014).

### Method Evaluation

#### Machine Learning

**Fig. 2:**
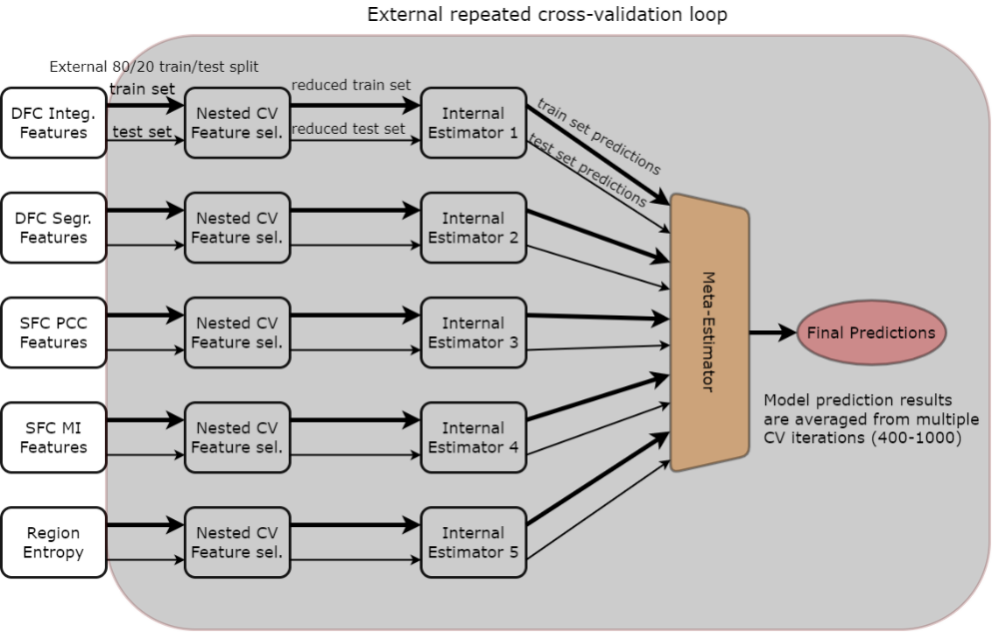
Decision-level machine learning fusion pipeline. The externally evaluated steps which were repeated for thousands of times through cross validation are enclosed within the gray area. Fixed steps, performed only once are placed on white background

Due to the very large number of total features (4(FC types) x 930(network metrics) + 232(regional entropy values) = 3952) as well as additional considerations discussed next, a decision-level machine learning fusion approach was followed. Apart from alleviating the problem of a high dimensional input, i.e., a very large number of features, decision-level fusion allows for a “parallel” treatment of different types of imaging metrics, producing “expert” models for each type, and fusing their decisions toward the final prediction. Initially, as shown in Fig. 2, feature sets from different methods are reduced individually, then each of the five models is trained independently (internal estimators 1–5) and, finally, the meta-estimator is trained only on lower-level model predictions. Here, internal estimator refers to a more traditional ML model (e.g., Random Forest or XGBoost), that produces predictions utilized for training and testing the meta-estimator. The external level estimator (gray background in Fig. 2) that is analyzed and evaluated formally, contains all internal estimators, meta-estimator, and feature selection models. To produce a final prediction on previously unseen data, internal estimators 1–5 each make their prediction for that participant and the meta-estimator having learned each individual model’s strengths and weaknesses is able to make a prediction based on class probability estimates alone.

In terms of feature selection and method validation, a consensus feature importance ranking-based, nested cross-validation methodology was implemented similar to Parvandeh et al. (2020), Zhong, Chalise, and He (2020), and our previous work (Simos et al., 2019; 2020). This choice was made to avoid overfitting and ensure a robust final model with conservative results and highly representative features for the comparison of interest. Consensus features were selected internally on 200 repeated, stratified 6-fold iterations performed on the training data based on highest feature importance rankings (produced by an ensemble-type classification model). Model performance metrics (accuracy, precision, sensitivity, specificity, F1 score, ROC AUC) were averaged over 1000 outer cross-validation iterations (repeated, stratified 5-fold CV). In each CV split or (external) iteration, the model steps enclosed in the gray area of Fig. 2 were repeated, including feature selection, internal-estimator model training, meta-estimator model training with internal estimator predictions, and, finally, testing on the 20% portion of unseen data. XGBoost (Chen and Guestrin, 2016), an implementation of regularized gradient boosted trees, was utilized as the base estimator for classification and feature selection models. Logistic Regression was chosen as the Meta-Estimator, as it is a relatively simple model able to be trained using the predictions of internal estimators and eventually learn the predictive intricacies of each feature set (Ho, Hull, and Srihari, 1994).

### Statistical Analysis

To support the interpretability and significance of ML results, independent sample t-tests were used to assess the statistical significance of group differences on each of the final features derived by the decision-fusion classification model. Control of familywise error rate across multiple comparisons was employed using the FDR (Benjamini and Hochberg, 1995) method and a false discovery rate q = 0.05. All statistical comparisons were performed in MATLAB.

Final features (i.e., FC/entropy indices) derived by the selection-classification-validation process and subsequently deemed significantly different between the two clinical populations were then evaluated for their potential associations with neuropsychological measures within the mTBI group. Pearson correlation coefficients were calculated between the patients’ functional indices (10 (features) x 5 (functional index types)) and their neuropsychological measures (12 in total). Correlation p-values were corrected for multiple comparisons with FDR for each feature.

## Results

### Neuropsychological and Conventional Imaging Findings

As shown in Supplementary Tables 1, small posttraumatic gliotic areas due to contusions (≤ 3 cm in extent; C) were found in 40.5% of the patients (in 7/37 patients in the left temporal lobe, in 7/37 patients in the right temporal lobe, in 7/37 patients in the right frontal lobe, and 8/37 patients in the left frontal lobe). Evidence of ≤ 3 chronic hemorrhagic DAIs was found in 27% of the patients (in 4/37, 1/37, 6/37, and 9/37 patients in the right temporal, left temporal, right frontal, or left frontal lobes, respectively).

Clinically significant depression or anxiety symptoms (according to the corresponding cutoff scores on the CESD and STAI scales) were noted in 21.6% and 48.6% of the patients, respectively (see Table 1). Deficits in the domains of episodic memory or attention control and executive function (as indicated by performance > 1.5 SD below the national norms on at least two relevant cognitive tasks) were present in 24.3% and 32.4% of the patients. On individual tests, patient average performance was below 1 SD from the population mean on Immediate (z=-1.10, SD = 1.1, p < 0.001) and Delayed Passage recall (z=-1.17, SD = 1.0, p < 0.001), Phonemic Verbal Fluency (z=-1.14, SD = 0.7, p < 0.001), and WAIS-IV Matrices (z=-1.01, SD = 1.1, p < 0.001). However, the presence of any of the aforementioned neuropsychiatric manifestations did not correlate with evidence of contusion or DAI in either frontal or temporal lobes (all Spearman ρ’s < 0.2, p = 0.2).

### Validation of DFC Brain State Identification

Initially, to examine the soundness of the DFC state identification process based on unsupervised learning (*k*-means clustering), as well as the validity of the integrated/segregated representative networks produced for each subject, a number of basic indices were evaluated. Mean participation coefficient was found significantly greater in the integrated state (p < 2×10^− 10^) and elevated modularity was found in the segregated state (p < 6×10^− 6^) as expected. The more pronounced modular structure is evident in the DFC networks from a representative participant in Supplementary Fig. 8. Again, as expected, small world propensity was found to be significantly greater in the segregated compared to the integrated state (p < 2×10^− 4^). Finally, silhouette values, a goodness of fit measure designed for clustering algorithms, indicated the optimal number of clusters to be 2 when a range of 2 to 7 clusters was tested (supplementary data). These measures are in line with previous adaptations of the DFC temporal state identification framework.

### Machine Learning Classification

The decision fusion approach combining several types of FC measures within a single robust Machine Learning model was modestly accurate in discriminating between mTBI and HC participants with balanced sensitivity (74%) and specificity (76%; Table 2).

**Table 2:** Machine Learning fusion classification performance (mean ± SD)

Accuracy	Precision	Sensitivity	Specificity	F1	ROC AUC
75 ± 9	77 ± 12	74 ± 14	76 ± 14	74 ± 10	75 ± 9

### Statistical Testing and Association of Imaging with Behavior

**Fig. 3:**
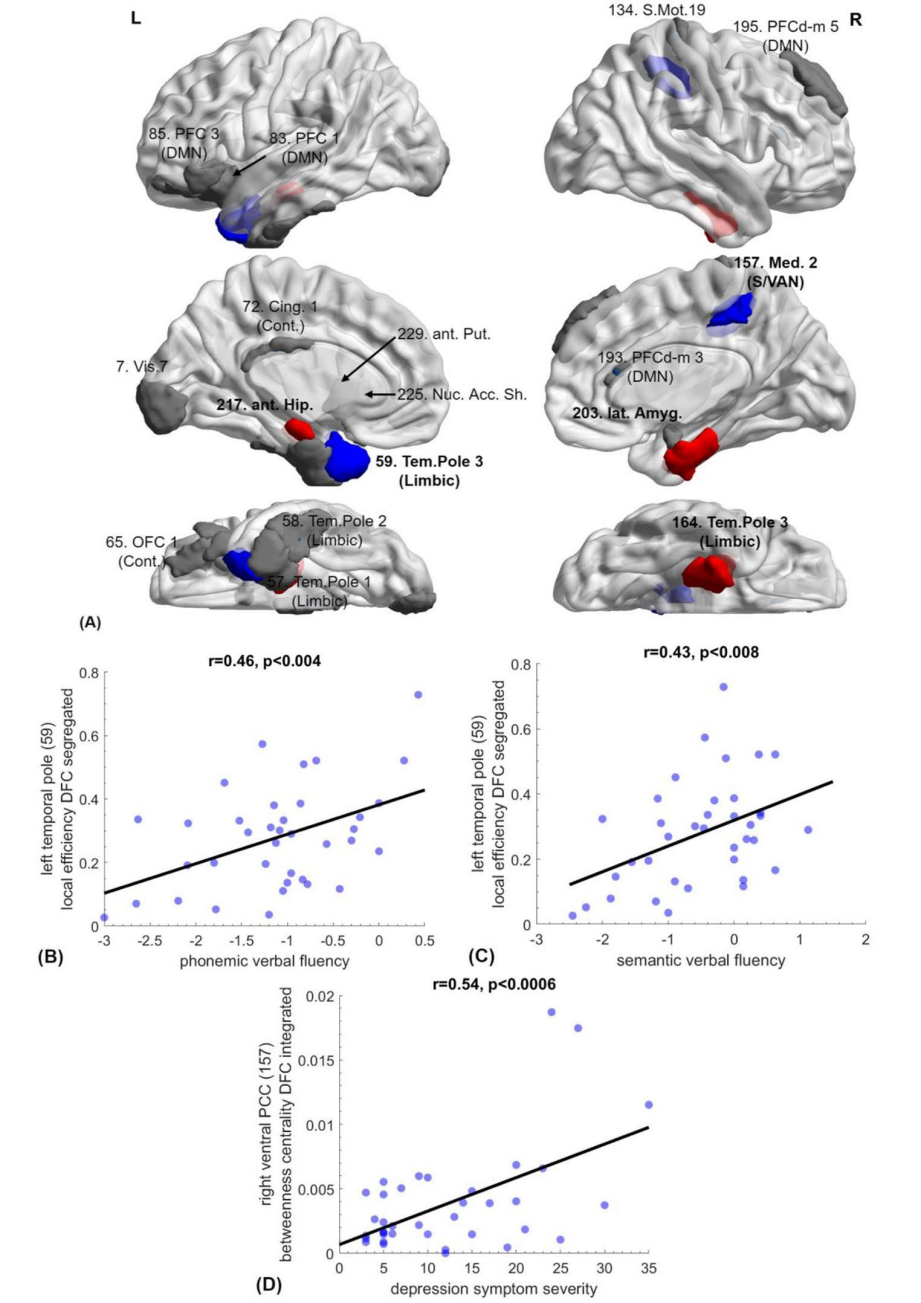
**(A) Areas displaying reduced functional connectivity in mTBI patients as compared to healthy controls**: 164: right temporal pole (on Prsn and MI SFC). 217: left anterior hippocampus (on centrality in both DFC states). 59: left temporal pole (LE-DFC), 157: right dorsal PCC (BC-DFC), 7: left secondary visual cortex (LE-SFC-MI), 57: left parahippocampal gyrus (LE-SFC-MI), 58: left inferior temporal gyrus (LE-SFC-MI), 72: left PCC (EC-DFC), 65/83/85: left ventrolateral prefrontal cortex (LE-SFC-MI), 134: right primary motor cortex (BC-SFC-Prsn), 193: right dorsal anterior cingulate cortex (LE-SFC-MI), 195: right dorsolateral prefrontal cortex (EC-DFC), 203: right lateral amygdala (EC-DFC), 225: left nucleus accumbens shell (EC-DFC), 229: left anterior putamen (DC-SFC-MI). Areas that featured in multiple FC indices are shown in red and areas where significant positive correlations were found with neuropsychological measures are shown in blue. **(B-D) Associations between FC metrics and cognitive/emotional status among mTBI patients (**FDR-corrected). **(B)** Left temporal pole LE with phonemic (r = 0.46, p < 0.004) and semantic (r = 0.43, p < 0.008) verbal fluency. **(C)** Right dorsal PCC BC with depression symptom severity (r = 0.54, p < 0.0006). Abbreviations; LE: local efficiency, BC: betweenness centrality, EC: eigenvector centrality, DC: degree centrality, MI: Mutual Information, Prsn: Pearson correlation, S/DFC: Static/Dynamic functional connectivity.

**Fig. 4:**
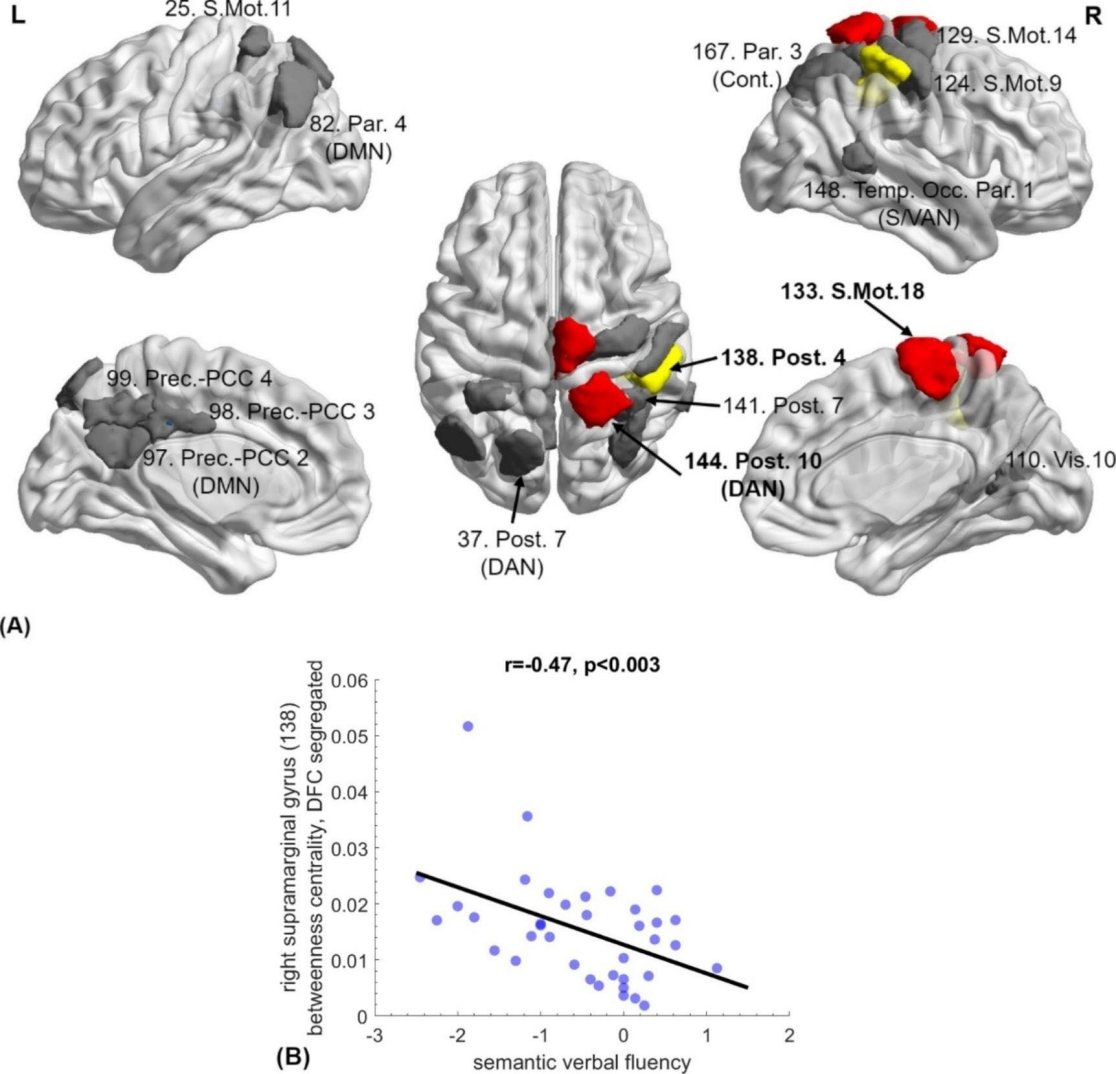
**(A) Areas displaying increased functional connectivity in mTBI patients as compared to healthy controls**: 133: right precentral gyrus (LE in both DFC states), 141/144: right superior parietal gyrus (centrality in both DFC states and SFC LE), 138: right supramarginal gyrus (BC-DFC), 25/37: left superior parietal (LE-SFC-Prsn), 82: left angular gyrus (LE-SFC-Prsn), 97/98/99: left ventral and dorsal PCC (LE-DFC), 110: right ventral PCC (LE-SFC-Prsn), 124: right primary somatosensory cortex (LE-SFC-MI), 129: right supplementary motor area (LE-DFC), 148/167: right angular gyrus (LE-DFC). Areas that featured in multiple FC indices are shown in red. **(B)** Betweenness centrality of the right supramarginal gyrus (138, shown in yellow in [A]) correlated negatively with semantic verbal fluency (r=-0.47, p < 0.003 FDR-corrected). Abbreviations; LE: local efficiency, BC: betweenness centrality, MI: Mutual Information, Prsn: Pearson correla?tion, S/DFC: Static/Dynamic functional connectivity.

**Fig. 5:**
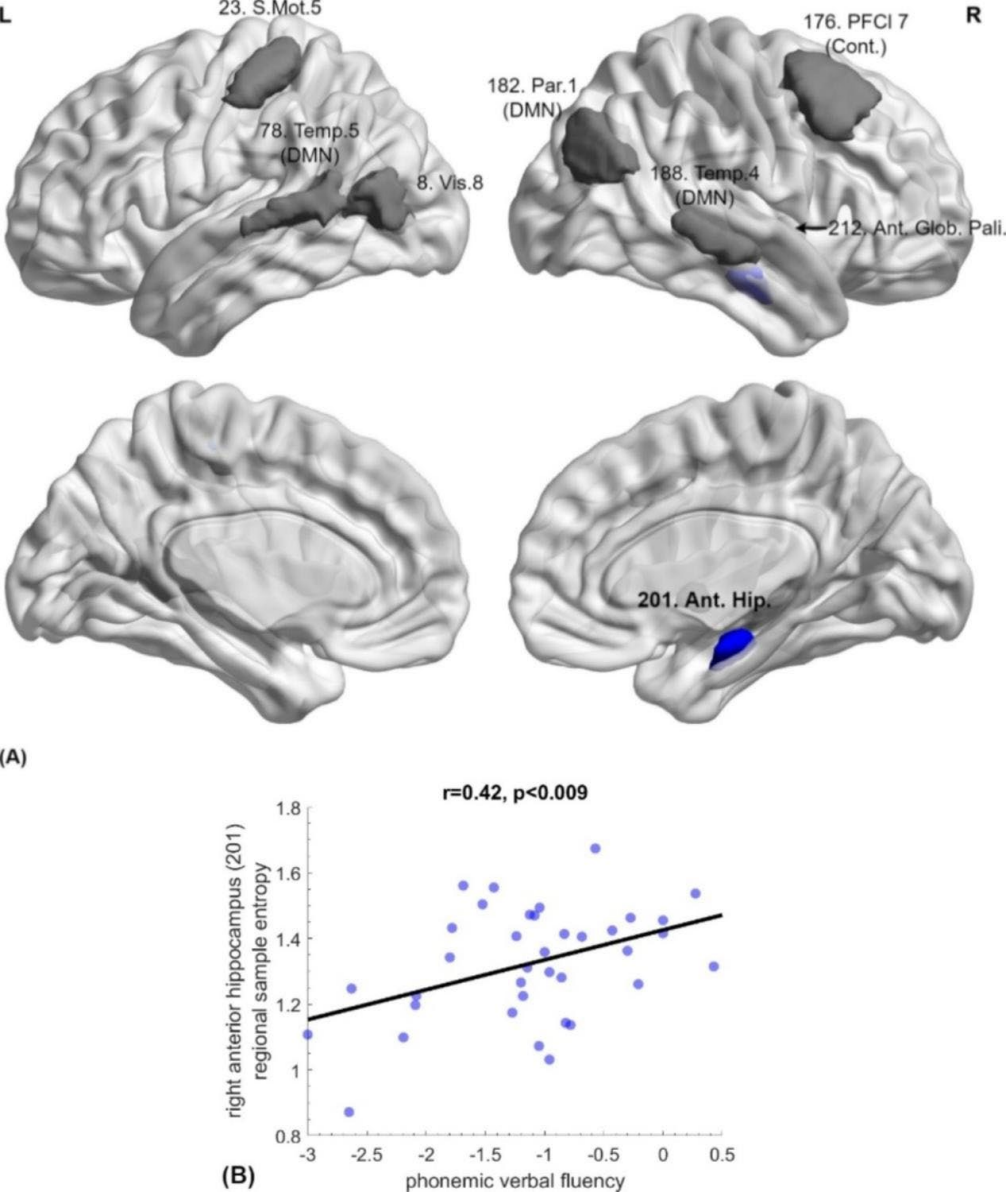
**(A) Decreased regional entropy in mTBI patients compared to healthy controls**: 201: right anterior hippocampus, 8: left cuneus, 23: primary somatosensory cortex, 78: left middle temporal gyrus, 176: pre-supplementary motor area, 182: right angular gyrus, 188: right middle temporal gyrus, 212: right anterior globus pallidus **(B)** Regional sample entropy of the right anterior hippocampus (shown in blue in [A]) correlated positively with phonemic verbal fluency (r = 0.42, p < 0.009 uncorrected)

As detailed in previous sections, regional functional indices that emerged as essential in differentiating the mTBI and HC clinical groups using the proposed machine learning pipeline, were subsequently statistically compared between the two groups. Out of a total of 50 highest-ranking features (10 from each feature set), the two groups differed significantly on 48 (q < 0.05, FDR-corrected). These included local efficiency values (46% of the selected features), centrality measures including betweenness, eigenvector and degree centrality (34%), and regional sample entropy values (20%). The regional functionality indices (graph metrics and sample entropy) presented in Figs. 3, 4 and 5 illustrate these features grouped according to the direction of group differences (mTBI > controls, or the opposite).

Associations of imaging and behavior were examined through correlations of the aforementioned significant regional functionality indices and patients’ neuropsychological test scores. Results are presented below and illustrated via scatterplots in Figs. 3, 4 and 5.

Temporal lobe limbic regions were among the most notable areas exhibiting *reduced* FC in mTBI patients: medial temporal pole (bilaterally), left anterior hippocampus, and right amygdala. A similar significant trend was noted in anterior and medial prefrontal regions (right dorsomedial, left ventromedial, and left orbital cortex) as well as in the dorsal posterior cingulate cortex (PCC). Evidence for temporal pole and anterior hippocampus hypo-connectivity was consistently found on both static (Prsn and MI) and dynamic FC measures (state centrality). Hypoconnectivity in mTBI was also found in the lateral portion of the temporal pole. A significant positive correlation between local efficiency in the DFC segregated state and both phonemic (r = 0.46, p = 0.004, 1-β = 0.92) and semantic verbal fluency (r = 0.43, p = 0.008, FDR-corrected, 1-β = 0.88) indicated that the degree of preserved function in this region was important to sustain verbal fluency capacity. Finally, dorsal PCC centrality correlated with depression symptom severity (CESD score, r = 0.54, p = 0.0006, FDR-corrected, 1-β = 0.98). All regions exhibiting reduced FC are shown in Fig. 3.

In addition, localized *increases* in FC (Fig. 4) were observed in several parietal regions in both hemispheres, including areas of the DAN, DMN, and somatomotor networks. Additional DMN regions that displayed hyperconnectivity were the left precuneus and dorsal PCC. Hyperconnectivity of this cluster of regions was supported by several complementary indices, such as local efficiency in the SFC-derived network and centrality in both DFC states. Both DFC states also corroborate the apparent increase in centrality of the right precentral gyrus. Importantly, hyperconnectivity of the right supramarginal gyrus (indexed by centrality in the DFC segregated state) correlated negatively with semantic verbal fluency (r=-0.47, p = 0. 003, FDR-corrected, 1-β = 0.93).

To ensure that reported increases in the functional role of these regions were not spurious due to reduced regional activation associated with mTBI, we examined regional entropy via the sample entropy values computed on regional timecourses. A slight decrease in entropy was detected as compared to the healthy control group in some region’s cases, which did not reach significance (p > 0.02 uncorrected). A few other regions, however, contributed through entropy indices to the machine learning classification between mTBI and healthy control groups (Fig. 5: all displaying reduced sample entropy in the mTBI group; p < 0.05, FDR-corrected). Among these regions, relatively lower sample entropy in the right anterior hippocampus was positively related to phonemic verbal fluency among mTBI patients (r = 0.42, p = 0.009, uncorrected, 1-β = 0.88). There were no significant correlations between connectivity or entropy indices and demographic variables or time post injury (p > 0.05 FDR-corrected). In view of the higher average educational attainment of the control group (by 2.2 years), we performed additional analyses to assess the potential effect of education level on the FC metrics within each group, without revealing significant correlations (FDR-corrected p > 0.05).

## Discussion

The present study set out to identify aberrant connectomic and dynamic features persisting into the chronic phase following injury in a clinical sample of mTBI patients. The novelty of the present work is threefold: **(a)** In using data-driven machine learning on static and dynamic FC features derived from person-specific analyses instead of a priori-defined or group-representative networks, **(b)** In examining the dynamic behavior of FC patterns to characterize patterns of reorganization in individual patient connectomes, and **(c)** In establishing associations of regional, static and dynamic FC indices with lingering cognitive and emotional difficulties.

### Functional Deficiencies in Chronic mTBI

Given the very limited previous research integrating indices of static and dynamic FC obtained in the chronic phase post-mTBI, only indirect comparisons with previous findings can be attempted. Hypoconnectivity of the temporal poles constitutes one of the most notable findings of the present study. Multiple measures (five features in total) from several very closely neighboring temporal pole subregions contributed to the final classification model. The importance of this region in mTBI is highlighted by static and dynamic connectivity (segregated state) and by connectivity networks derived by Prsn and MI. Nodal degree, a measure of centrality, and local efficiency, revealed similar trends reflecting an overall reduced functional role of this region as a functional hub in varying network scales. While this region is known to be susceptible to contusion injury following mTBI caused by direct impact on the cranium, and may present evidence of structural connectivity changes following mTBI (Van Der Horn et al., 2017), there is very limited evidence of temporal pole FC abnormalities using rs-fMRI in the literature. Further highlighting the region’s importance in mTBI, associations were detected between hypoconnectivity of the left temporal pole and verbal fluency (semantic and phonemic). Importantly, average verbal fluency scores of the present sample of mTBI patients were significantly below age- and education-adjusted population norms (Table 1). This finding is in agreement with the moderate-to-large effect size for persisting verbal fluency impairment in the semi-acute/chronic phase post mTBI reported by Belanger and colleagues (2005). Corroborating results have been also reported by a study correlating voxel intensities in the temporal pole, extending more posteriorly into the inferior temporal region, with cross-modal integration performance in brain damaged patients (Taylor, Stamatakis, and Tyler, 2009). Taken together, these findings suggest that preserved functional connectivity of the left temporopolar cortex is crucial for the maintenance (or recovery) of lexical/semantic storage and retrieval capacity even in mTBI. The role of temporopolar cortex, especially in the left hemisphere, in the organization and storage of lexical/semantic representations is supported by several lines of evidence (for reviews see Binder et al., 2009; Hickok and Poeppel, 2007; Patterson, Nestor, and Rogers, 2007; Price, 2012). This accumulating evidence is consistent with a recent notion that the temporopolar neocortex functions as a multimodal convergence hub that plays a key role in semantic control which is in turn crucial for efficient performance on category and phonemic verbal fluency tasks (Ralph et al., 2016).

There was also hypoconnectivity (indexed by reduced centrality in the DFC integrated state) of the dorsal PCC, in agreement with Vergara et al. (2016). Although the precise role of the PCC for depression symptoms is unclear, there have been reports of reduced FC in patients with major depressive disorder in this region (Yang et al., 2016). Furthermore, in a large-scale rs-fMRI FC study focusing on depression-related biomarkers, Drysdale and colleagues (2017) identified the PCC as one of the key regions with common aberrant FC characteristics across the four patient clusters they studied, as defined on the basis of connectomic features.

Hypoconnectivity of key limbic (amygdala and anterior hippocampus) and frontolimbic structures (vmPFC, ventral OFC) were also noted in the present study, although the degree of aberrant FC in these regions did not correlate significantly with emotional manifestations (anxiety/depression symptoms). Alterations in limbic and frontolimbic connectivity may indicate subtler changes in emotion regulation, but they could be linked to impulsivity and irritability symptoms, and potential future psychological difficulties (which were not measured in the present study).

Finally, reduced complexity of the BOLD timecourses in the left anterior hippocampus was tentatively associated to lower phonemic verbal fluency capacity among mTBI patients. This finding highlights the central role of hippocampal functional integrity to enable verbal fluency, in agreement with literature describing its role for simple and complex memory, also a prerequisite for effective coping mechanisms. Although medial temporal structures are traditionally linked to episodic memory, there have been reports of impaired semantic and/or phonemic fluency impairment in medial temporal amnesia (Greenberg et al., 2009) and fMRI data suggest that medial temporal involvement in these tasks is related to the autobiographic component of certain lexical/semantic representations (Sheldon and Moscovitch, 2012).

### Increased FC in Chronic mTBI

Several posterior regions of the Dorsal Attention Network (DAN) exhibited elevated local efficiency and centrality (node degree or eigenvector centrality), indicated by static and dynamic measures (Prsn and MI connectivity metrics). Our observations consolidate previous findings of increased (static) connectivity of the DAN (Champagne et al., 2020) in chronic mTBI. The putative enhanced functional role (as indexed by local efficiency and node degree) of several regions of the somatomotor network is also in line with the reported increase in connectivity in somatomotor and DAN regions including the precentral gyrus and inferior parietal lobule in semi-acute mTBI patients (Mayer et al., 2014). Similarly, hyperconnectivity of the posterior precuneus noted in the present study was also reporter by Vergara et al. in semi-acute mTBI patients using SFC (Vergara et al., 2016). They also found increased DFC in the paracentral/precentral gyrus (SMA) which is extended by our results of hyperconnectivity (as indicated by both SFC and DFC indices). The heightened functional role of visual and ventral attention network regions is in line with increased (static) connectivity reported in chronic mTBI patients (Champagne et al., 2020), and the elevated connectivity of visual cortex in sub-acute patients through DFC (Mayer et al., 2014). Although not directly addressed by our data, we speculate that hyperconnectivity of the DAN may reflect a state of hypervigilance in an attempt to compensate for persistently reduced limbic and frontolimbic connectivity.

It should be noted that hyperconnectivity in these regions was indexed mainly by centrality, reflecting the extent to which the regions operate as important connecting hubs within the large underlying networks. In spite of the expected individual variability across patients, these dorsal and medial parietal regions are likely higher order network hub regions which had been recruited to preserve function after TBI. However, the effectiveness of this putative network reorganization is not particularly effective, given the negative associations we found between FC indices and verbal fluency scores.

### Significance of Findings

Due to substantial inter-patient variability in trauma location/direction, type and force, regions that show aberrant FC are expected to vary across patients. To address this issue in the classification problem we computed indices of connectivity that preserved individual differences in connectome profiles. Even though key regions of significance derived for the current clinical group are potentially higher-order major network hubs of regional signaling, their value as potential biomarkers is supported by their contribution to classification performance and significant associations with cognitive and emotional measures.

Delving deeper into mTBI-specific connectomic characteristics, most of the functional measures that were increased in the mTBI group were measures of local efficiency indicating functional segregation, a finding corroborated by the predominance of segregated state features in mTBI patients. Thus, several indices point to an overall more segregated network in this group. Conversely, the set of FC features that were found to be increased in the control group include both local efficiency and centrality measures, with a predominance of integrated state measures.

Another aspect of the present study with potential clinical implications concerns aberrant FC patterns involving anterior temporal regions, which mapped consistently onto verbal fluency performance. Language functions have received less attention in previous studies on cognitive recovery following mTBI, focussing mainly on verbal fluency (Bell et al., 1999; Konrad et al., 2011; Vanderploeg, Curtiss, and Belanger, 2005). The latter is commonly assessed through word generation tasks based on semantic or phonemic criteria, performance on which relies on the availability of intact lexical and phonological representations as well as processing speed and various executive processes (Henry and Crawford, 2004). In view of the purported predictive value of verbal fluency in the prognosis of recovery from TBI (across severity types; Ponsford, Draper, and Schönberger, 2008), it remains to be seen if these functional connectivity indices would also emerge as significant prognostic factors for successful cognitive recovery following mTBI.

Additional relevant characteristics of the present patient sample further support the validity of results. Firstly, none of the patients were involved in litigation concerning the injury or seeking compensation for incurred damages, thus malingering as a factor contributing to their test scores was not likely. Secondly, their neurocognitive status largely reflected the “natural” course of illness recovery, not affected by systematic neuropsychiatric interventions. These characteristics render the present sample suitable to assess the chronic impact of mTBI given the, at least modest, effect of suspected malingering (Ross, Putnam, and Adams, 2006), and the documented impact of cognitive (Mahncke et al., 2021) and physical rehabilitation (Snyder et al., 2021) upon cognitive recovery.

### Study Limitations and Future Work

A key limitation of the current study pertains to the number of participants. Apart from allowing increased generalizability and more robust statistical results, the nested CV scheme implemented, which splits the dataset twice (externally and internally), would certainly benefit from a larger sample size. The observed sample standard deviation of the classification performance metrics is also expected to improve with a larger sample size.

Moreover, interpretation of our findings should take into account that the present patient sample was recruited from the registry of a neurosurgery clinic. This may account for the high frequency of even small posttraumatic structural lesions visible on conventional MRI (present in 40% of cases) and the presence of post-acute cognitive deficits in a substantial proportion of patients, whereas the presence of reliably documented deficits in non-clinical, civilian samples in the chronic phase post mTBI has been questioned (Dikmen et al., 2009). It should be noted, however, that the frequency of significant depressive symptomatology in our sample is comparable to that reported by other studies (22%) compared to depression rates of 10–20% in other mTBI samples (Rapoport, 2012; Shoumitro et al., 1999). Additional assessments, not included in the present study, such as other scales of everyday behavioral changes, would help complete the clinical picture of mTBI patients, and potentially reveal further associations with FC metrics.

A further notable limitation of the current study is that neuropsychological and emotional symptom scores were not available on HC participants as they would facilitate interpretation of the correlational analysis results performed in the mTBI group (provided that sufficient variability in these scores were present among healthy adult volunteers). Therefore, the specificity of the significant associations between FC indices and test scores for mTBI is not conclusive.

Inevitably, trade-offs exist between different methods to identify time-resolved functional connectivity patterns, and a large variety of alternatives have been developed in recent years (Fukushima, Betzel, He, de Reus et al., 2018; Lurie et al., 2020). The “cartographic profile” approach of Shine and colleagues (2016), which we adopted here, has been used in multiple previous studies, including studies with patients suffering from severe brain injury (Luppi et al., 2019). A potential alternative would be adding predominately small-world and rich-club states, as the notion of easily distinguishable integrated-segregated network states, although relatively straightforward, may not be truly representative of the resting functional brain organization of every individual and population. Additionally, the decision-level machine learning fusion approach utilized in the present work can be complemented by additional sets of data to improve prediction accuracy, including behavioral measures (which were not presently available in the control group), structural connectivity indices (diffusion MRI combined with rs-fMRI as suggested by Sharp and Leech, 2014), metrics of regional perfusion dynamics (cerebral blood flow indices) (Champagne et al., 2020; Wang et al., 2019) or blood-based biomarkers (Posti and Tenovuo, 2022). Finally, the potential benefits from increased temporal and spatial resolution by using longer BOLD time series obtained in a 3T scanner are worth mentioning. We attempted to mitigate these limitations by implementing an ROI-based approach, which does not make claims of fine-grained connectivity maps. However, increased SNR and especially longer timeseries achieved by lower TR values could potentially benefit the analysis, especially due to the dynamic nature of some of the methods applied.

## Conclusion

The present study advances previous reports on aberrant FC in chronic mTBI patients by employing a robust machine learning approach toward feature selection and cross-validation complemented by conventional assessment of group differences and associations with neuropsychological measures. Furthermore, analyses on the entropy of the regional hemodynamic signals confirmed that FC changes were not associated with reduced activation complexity, contributing to elucidate the links between brain dynamics and connectomics in mTBI. The machine learning classification results reported here can be built upon toward forming an effective diagnostic model. Notably, these classification metrics were obtained via a very conservative consensus-based feature selection in a nested cross validation scheme, ensuring, as much as possible, real-world model performance. Such model designs are not yet commonplace in neuroimaging studies, making direct comparisons challenging. Due to the utilized evaluation scheme, the reported results should be indicative of model performance if tested with new patient data, and we auspicate that future work will follow our example to make this robust approach more widespread.

## Electronic Supplementary Material

Below is the link to the electronic supplementary material.

Supplementary Material 1

## Data Availability

The data that support the findings of this study are available from the corresponding author, upon reasonable request. Details on code and toolboxes used in the analyses are given in the supplementary data.

## Abbreviations

mTBI: mild Traumatic Brain Injury
rs-fMRI: resting-state functional Magnetic Resonance Imaging
GCS: Glasgow Comma Scale
ML: Machine Learning
S/D FC: Static/Dynamic Functional Connectivity
ROI: Region Of Interest
FCG: Functional Connectivity Graph
OMST: Orthogonal Minimum Spanning Trees
CV: Cross Validation.
