# Supplementary material for: Chronic Mild Traumatic Brain Injury: Aberrant Static and Dynamic Connectomic Features Identified Through Machine Learning Model Fusion
Source: Neuroinformatics. 2022 Dec 2;21(2):427–42. doi: 10.1007/s12021-022-09615-1 (PMC10085953; doi:10.1007/s12021-022-09615-1)
Supplement: Supplementary file 1 — Supplementary Material 1 [file 12021_2022_9615_MOESM1_ESM.docx]

**Supplementary material**

**Chronic mild traumatic brain injury:** **Aberrant static and dynamic connectomic features identified through machine learning model fusion**

Nicholas J. Simos,1^* Katina Manolitsi,^2,5*^ Andrea I. Luppi,^3,4^ Antonios Kagialis,^5^ Marios Antonakakis,^6^ Michalis Zervakis,^6^ Despina Antypa,^5^ Eleftherios Kavroulakis,^7^ Thomas G. Maris,^1,7^ Antonios Vakis,^2^ Emmanuel A. Stamatakis,3,4^ and Efrosini Papadaki^1,7^

**Author affiliations:**

1 Computational Bio-Medicine Laboratory, Institute of Computer Science, Foundation for Research and Technology–Hellas, 70013 Heraklion, Greece

2 Department of Neurosurgery, School of Medicine & University Hospital of Heraklion, University of Crete, Crete, Greece

3 Division of Anaesthesia, School of Clinical Medicine, University of Cambridge, Addenbrooke’s Hospital, Hills Rd, CB2 0SP Cambridge, UK

4 Department of Clinical Neurosciences, School of Clinical Medicine, University of Cambridge, Addenbrooke’s Hospital, Hills Rd, CB2 0SP Cambridge, UK

5 Department of Psychiatry, School of Medicine & University Hospital of Heraklion, University of Crete, Crete, Greece

6 Digital Image and Signal Processing Laboratory, School of Electrical and Computer Engineering, Technical University of Crete, Chania, Greece.

7 Department of Radiology, School of Medicine & University Hospital of Heraklion, University of Crete, Crete, Greece

^Correspondence to: Emmanuel A Stamatakis PhD

Full address:

Division of Anaesthesia

University Of Cambridge

Box 93, Addenbrooke's Hospital

Hills Road

Cambridge, CB2 0QQ

U.K.
E-mail: [eas46@cam.ac.uk](mailto:eas46@cam.ac.uk)

^Correspondence to: Nicholas J. Simos

Full address:

Computational Bio-Medicine Laboratory

Institute of Computer Science

Foundation for Research and Technology–Hellas (FORTH)

Nikolaou Plastira 100

P.O Box 1385

Vassilika Vouton, 70013 Heraklion, Crete, Greece

E-mail: nicholasjohnsimos@gmail.com

*Authors NJS and KM had equal contribution to this work

# Materials and methods

## Functional network measures

In the current study, weighted-undirected networks were used in all analysis steps, based on Prsn or MI indices (Rubinov and Sporns 2010). Some basic notation used for the calculation of several graph metrics below:

- ***N****:* all the nodes of the network
- ***n***: the number of nodes
- ***v***: sum of the network’s connections, $\boldsymbol{v}= \sum_{i,j \in N} w_{ij}$
- ***w_ij_****:* the edge weight between nodes *i* and *j*.
- ***a_ij_*** = 1 when a connection exists between *i,j*, 0 otherwise.
- The **shortest path** ***d_ij_*** between nodes *i,j* is the path connecting the two nodes with the least sum of distances (maximum sum of strengths).

Most metrics operate on distance weighted networks, thus all formulas and discussion of graph metrics refers to distance weighting. A transformation of 1/FCG is used in all cases when needed, taking the edge-wise multiplicative inverse.

### Characteristic Path Length

The characteristic path length of a given graph (L) is the average shortest path between all pairs of nodes. High values indicate a global trend of connections/paths with low cost (short length). It is thus a global measure of functional integration. In general, integrated networks exhibit globally strong connectedness that is controlled by hub nodes. A more diffuse connectedness is expected in such networks.

$$L= \frac{1}{n}\sum_{i\in N} \frac{\sum_{i\in N,j\neq i} d_{ij}}{n-1}$$

### Global Efficiency

Global Efficiency (GE), calculated as the average inverse shortest path length, is inversely related to the characteristic path length. The interpretations and uses of global efficiency are very similar to those of characteristic path length but with inverse value ranges. Lower values of characteristic path length indicate more efficient communication overall, high values of global efficiency indicate the same. Additionally, the characteristic path length is mostly influenced by longer paths while the opposite is true for global efficiency, this also makes global efficiency better fitted for judging the communication structure in disconnected graphs. In these cases, the calculated efficiency value is zero.

$$GE= \frac{1}{n}\sum_{i\in N} \frac{\sum_{i\in N,j\neq i} \frac{1}{d_{ij}}}{n-1}$$

### Local Efficiency

Higher values of Local Efficiency (LE) (or clustering coefficient) indicate better communication efficiency among the nodes of an immediate local community. Local efficiency is equivalent to global efficiency calculated on the neighborhood of a node. It is also quite similar to the clustering coefficient as it is described below, for this reason typically one of the two measures is used per application. The inverse shortest path length calculated on the subgraph of all immediate neighbors of a particular node is then normalized by the possible connections that could exist among those nodes. In general terms, it encapsulates whether a system is more immune to faults by quantifying the efficiency of communication between a node and its neighbors if it were removed from the network.

$$E_{LOC}= \frac{1}{n}\sum_{i\in N} \frac{\sum_{i\in N,j\neq i} {(w_{ij}w_{ih}\frac{1}{d_{ij(N_{i})}})}^{1/3}}{k_{i}(k_{i}-1)}$$

### Clustering Coefficient

The quantification of segregation via increased clustering in complex networks usually revolves around the prevalence of triangles. The ratio of neighbors around a particular node that are also neighbors with each other, calculated through a fraction of triangles, is known as the binary clustering coefficient (C) (Watts and Strogatz 1998). In the weighted graph case, conceptually and computationally similar to local efficiency, the clustering coefficient measures the degree of information segregation at a given node’s neighborhood. Segregated networks are typically characterized by more clearly formed local communities, disconnected from each other and with strong internal associations.

$C= \frac{1}{n}\sum_{i\in N} \frac{2t_{i}}{k_{i}(k_{i}-1)}$

*Where ti is the geometric mean of triangles around the node I according to the following formula:*

$t_{i}=\frac{1}{2}\sum_{j,h\in N} {(w_{ij}w_{ih}w_{jh})}^{\frac{1}{3}}$

### Small-Worldness

Networks that exhibit increased local clustering combined with short links that connect these local communities with distinct parts of the network are referred to as ‘small world’ networks. Small-world networks manage to maintain good overall connectedness (relatively high Global Efficiency) without most nodes having an increased number of connections. They represent a balance between the high global efficiency of random networks and the high local efficiency found in lattice networks (see Supplementary Figure 1). The straightforward approach to calculate small-worldness is to obtain the ratio of the standardized clustering coefficient to standardized characteristic path length. The weighted clustering coefficient strongly resembles the weighted local efficiency, while global efficiency is the inverse of the characteristic path length. Standardization can be achieved by dividing each metric by the mean values of those obtained from “random” networks. The random networks can be derived from random rewirings of the initial network. Small-worldness values obtained from the given formula are often much greater than one.

$$SW=\frac{C/C_{rand}}{L/ L_{rand}}$$

*Where:*

*C: Clustering coefficient, C_rand_: Clustering coefficient of random network
L: Characteristic path length, L_rand_: Characteristic path length of random network*

Conceptually, small-world networks can be considered as a balance between local and global processing (Bassett and Bullmore 2017). Additionally, more prominent small-world characteristics tend to indicate efficiency-optimality of information capacity (Barttfelda et al. 2015).


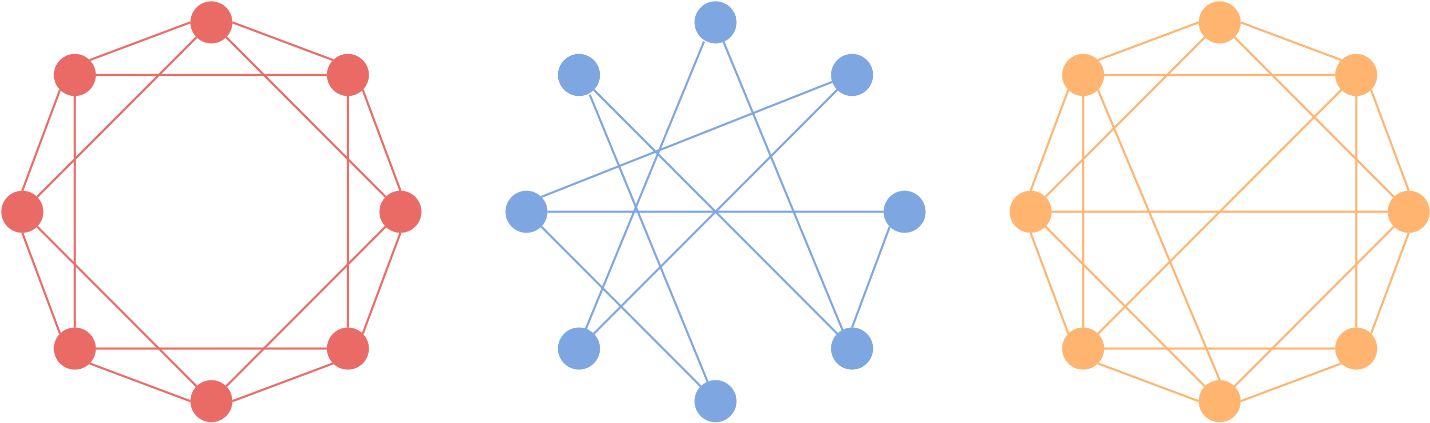


Supplementary figure 1 – lattice network - random network - small world network

### Small-World Propensity

The small-worldness measure defined above has a few inherent drawbacks. Namely, it is dependent on a network’s density without taking into account the edge weights which significantly limits the measure’s ability to be compared across different network topologies of varying densities and overall wiring weights. This issue is particularly problematic when comparing brain networks from different individuals, conditions, repeated scans etc. For these reasons, an improved metric was formulated, namely, small-world propensity (SWP) (Muldoon, Bridgeford, and Bassett 2016) which quantifies the small-world characteristics of a given network while taking into account variations in density. The deviation of the given network’s clustering coefficient and characteristic path length is measured against those calculated on lattice and random networks. The random and lattice networks created for these comparisons retain the same degree distributions as the original. Finally, the SWP produces values ranging for 0 to 1, different from small worldness which can be higher than 1. The sensitivity of SWP has also been corroborated by (Luppi, Carhart-Harris, et al. 2021), in a similar approach as the one adopted in the present study.

$$SWP=1- \sqrt{\frac{\Delta_{c}^{2}+\Delta_{L}^{2}}{2}}$$

*Where:*

*C_obs_: Observed clustering coefficient,*

*L_obs_: Observed characteristic path length
C_rand_: Random network clustering coefficient,*

*L_rand_: Random network characteristic path length*

*C_latt_: Lattice network clustering coefficient,*

*L_latt_: Lattice network characteristic path length
Δ_C_, Δ_L_ are calculated as the fractional deviation of the network’s actual clustering coefficient, characteristic path length against those of a lattice and random network according to the following formulas:*

$\Delta_{c}=\frac{C_{latt}-C_{obs}}{C_{latt}-C_{rand}}$*,* $, \Delta_{L}=\frac{L_{obs}-L_{rand}}{L_{latt}-L_{rand}}$

### Degree

Functional centrality metrics attempt to encapsulate node importance in a single nodal graph measure. A node predominately acting as a network hub, interacting with many other nodes and facilitating integrative network behavior should correspond to increased values of centrality. Node degree (DEG) is the simplest measure of centrality, revealing the number of nodes that a given node interacts with (or overall strength of interactions). Binary node degree (*k^b^*) simply counts a particular node’s connections, while weighted degree (*k^w^*) sums over that node’s connection weights with all other nodes.

$$k_{i}^{b}= \sum_{j \in N} a_{ij} ,k_{i}^{w}= \sum_{j \in N} w_{ij}$$

### Betweenness Centrality

Betweenness Centrality (BC) attempts to measure node importance from the number of strong connections a given node is located between and thus helps to mediate. It is formally calculated as the number of shortest paths passing through a particular node divided by the total number of shortest paths of the network.

$${BC}_{i}= \frac{1}{(n-1)(n-2)}\sum_{\begin{aligned} h,j \in N \\ h\neq j,h\neq i,j\neq i \end{aligned}} \frac{{sp}_{hj}(i)}{{sp}_{hj}}$$

*Where:*

*sp: The number of shortest paths
sp_hj_: The number of shortest paths between nodes h and j
sp_hj_(i): Shortest paths between nodes h and j that cross over i.*

### Eigenvector Centrality

Eigenvector Centrality (EC) is a self-referential measure of centrality i.e., a node with high values of EC must be connected to other nodes with increased EC. This nodal centrality measure’s values are computed as eigenvector elements, corresponding to the highest eigenvalue of the adjacency matrix. Quite differently from node degree, EC (and BC), do not rely on the mere number of connections a node possesses but the ‘importance’ of its connections which in turn lead to higher values of centrality and importance. BC accomplishes this by incorporating the number of important connections in the calculated measure, while EC attempts to quantify node importance, i.e., relationships with more ‘important’ nodes increases that particular node’s importance/centrality.

### Functional Modules – Modularity

Often in networks, groups of strongly connected regions may emerge, exhibiting only weak associations with other nodes or groups. These strongly interconnected subcomponents of the original network are usually referred to as ‘modules’ or ‘communities’ (Newman 2004). The “weak connections” of modules towards other modules can also be negative correlations, as in the case of the Prsn that provides signed values. The detection of such clusters in complex brain networks provides important insight of the internal structure of the network. Additionally, the quantification of decomposability or ‘modularity’ can also be a significant condition or disease-related biomarker indicating a generalized dysfunction in connectedness. Modularity can be included in the category of functional segregation measures, as exhibiting a prevalence of isolated communities is considered predominately segregated behavior.

$$Q= \frac{1}{v} \sum_{ij\in N} (w_{ij}-\frac{k_{i}k_{j}}{v})\delta_{M_{i}M_{j}}$$

This modularity calculation can be interpreted as the average difference of actual within module connections, represented by *w_ij_* and within module connections expected to be present by chance.

### Assignment of Nodes to Modules: Louvain

Building upon the notion of functional modules and network decomposability, methods have been developed for the detection of such community-type structure. In contrast with all other network measures discussed here, the assignment of nodes to modules utilizes optimization and is therefore not computed directly. The latter is implemented via the Louvain greedy algorithm, where different possible partitions are evaluated and the “optimal” is chosen by maximizing the modularity function *Q*. When working with strictly positive-weighted networks as in the case of MI FCGs, the modularity calculation described above is used. Furthermore, as previously mentioned, connection sign can be utilized in the module assignment process, based on the notion that modules can exhibit anticorrelations among each other. Most algorithms treat negative and positive weights symmetrically, something problematic to justify and understand. Regarding the underlying neuronal network, negative connections are not typically considered as of high importance as positive. The main structures under study, the modules themselves, should be mainly defined by the strength of the positive connections existing between their nodes. Negative correlations should affect the network’s modular characterization more than simply weak connections but definitely not as importantly as (strong) positive connections. This is also emphasized in (Rubinov and Sporns 2011), were partitions obtained with high *Q^-^*are found less optimal than high *Q^+^* partitions, *Q^+/-^* referring to the modularity function for positive and negative weights accordingly.

The asymmetric treatment of positive and negative weights in the *Q^*^* function proposed Rubinov and Sporns (Rubinov and Sporns 2011), leads to desirable behavior in terms of *Q^*^*’s output. Firstly, an increase in positive connections reduces the effect of negative connections, while an increase in negative connections does not lessen the effect of positive connections on the partition’s computed modularity value. Finally, in a network with an equiprobable distribution of positive and negative weights, negative connections will affect the modularity value only half as much as the positive.

$$Q^{*} = Q^{+}+\frac{v^{-}}{v^{+}+ v^{-}}Q^{-} = \frac{1}{v^{+}}\sum_{ij} ({w_{ij}}^{+}-{e_{ij}}^{+})\delta_{M_{i}M_{j}}- \frac{1}{v^{+}+ v^{-}}({w_{ij}}^{-}-{e_{ij}}^{-})\delta_{M_{i}M_{j}}$$

*Where:*

*Q^*^, Q^+^, Q^-^: Modularity values for asymmetric treatment of positive & negative, positive, negative weighted networks accordingly.
$\delta_{M_{i}M_{j}}$: 1 when I and j are in the same module or 0 otherwise.
e_ij_^±^: The weight of an edge divided by the graphs total weight v^±^ given by the following formula:* $e_{ij}^{\pm}= \frac{k_{i}^{\pm}k_{j}^{\pm}}{v^{\pm}}$

Although many implementations make claims of reproducibility, in practice, a significant amount of stochasticity seems to exist in the results, demanding additional steps to ensure stability. A much needed step towards this direction is proposed by Rubinov, co-creator of the Brain Connectivity Toolbox (BCT), which includes the implementation of Louvain’s algorithm (Rubinov and Sporns 2011). This entails continuously calculating new module partitions while the modularity increases, stopping only when the change in modularity is minuscule (e.g., less than 0.00001). Similar iteration procedures have been proposed, repeating the module assignments 500 times and retaining the consensus assignment (Shine et al. 2016). A combination of both was implemented in the current study, externally iterated 100 times and internally until the modularity no longer increases. Externally, the assignment with maximized modularity was selected as the optimal partition. This simply ensures that the assignments are repeated at least one hundred times and the most modular selected, somewhat balancing the substantial stochasticity observed in consecutive runs.

Another potential source of inconsistencies in the results can be due to the so called “degenerate” partitions: partitions with similar (high) values of modularity, which may seem to “alternate” in appearance during calculation (Rubinov and Sporns 2011). An argument is made that in some cases degeneracy may be able to provide insightful results when multiple partitions are studied. This optional analytic step was not possible in the present study as it was not compatible with other parts of analysis.

### Participation Coefficient

The Participation Coefficient (PC) aims at measuring a node’s intermodular connections. In this manner, it reveals whether a particular node acts as a connector hub mediating global integration as illustrated in Supplementary Figure 2. The colored, intermodular edges are the main point under study by the PC. Its calculation includes the ratio of degree values towards other modules by that of connections towards all nodes.

$${PC}_{i}=1-\sum_{m\in M} \left( \frac{k_{i}^{w}(m)}{k_{i}^{w}} \right)^{2}$$

*Where:*

*M: the set of modules identified using the Louvain algorithm discussed above
k_i_(m): the number of links between the node i and all the nodes of module m.*


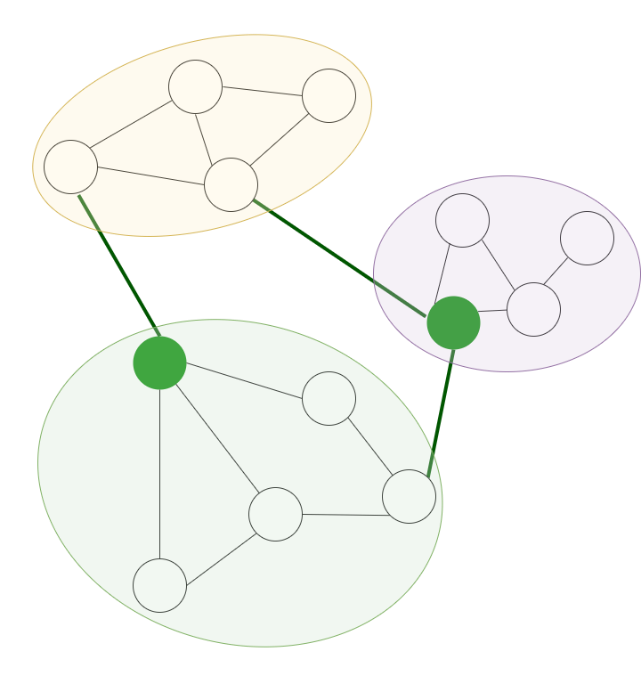
Supplementary figure 2 – Participation coefficient

### Within-Module Degree Z-Score

As illustrated in Supplementary Figure 3, within-module degree z-score attempts to describe a node’s intramodular degree of connectedness. It does so by normalizing weighted degree metric by means of the well-known z-score, subtracting the mean and dividing by the standard deviation. In this manner, the measure captures the strength of membership of a given node to its assigned module.

$$z_{i}= \frac{k_{i}^{w}\left( m_{i} \right)- \bar{k}_{i}^{w}(m_{i})}{\sigma^{k^{w}\left( m_{i} \right)}}$$

*Where:*

*m_i_: The module containing node i
k_i_(m_i_): The within-module degree of i
k bar represents the mean of the within-module degree distribution, while σ the standard deviation*


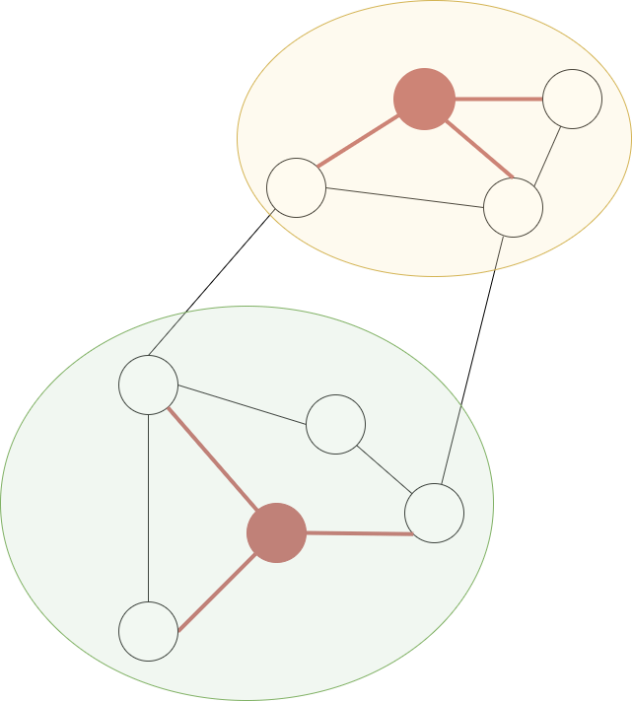


Supplementary figure 3 - within-module degree z-score

## Graph reduction–Orthogonal Minimum Spanning Trees (OMST)

Several topological graph filtering schemes have been proposed, based on the optimal detection of significant subnetworks and maintaining relevant structure rather than focusing on connectivity strength alone. For instance, Minimum Spanning Trees (MST) (Saba et al. 2019), maintains all network nodes with the “optimal” path between them. The ‘tree’ in minimum spanning tree refers to the acyclic form of the paths created, meaning there are no loops in the final network. The absence of loops also leads the tree to include *n*-1 edges exactly, where *n* the number of nodes. ‘Minimum’ refers to the minimization of total edge weight. The main drawback of MST is that in some instances the tree includes weak connections in order to satisfy the main criteria. Additionally, the solutions can often be considered too sparse, including only *n*-1 edges/connections, potentially too few for capturing complex interactions between brain regions (Antonakakis et al. 2016).

The OMST was introduced to address these issues (Dimitriadis et al. 2018; Dimitriadis, Antonakakis, et al. 2017; Dimitriadis, Salis, et al. 2017; Luppi, Gellersen, et al. 2021) The ‘O’ in OMST refers to the orthogonality between trees, a differentiating aspect from MST being, the creation of multiple trees that do not share common edges, instead of a single tree connecting all graph nodes. Multiple MSTs are formed, and the trees characterized by the most efficient information transfer are retained. The graph measure of global efficiency is utilized for quantifying this aspect of each MST, while overall wiring cost is also minimized. Cost refers to the ratio of the total edge weight divided by the total edge weight of the unreduced fully weighted graph. Multiple orthogonal (without overlapping edges) MSTs are combined, and the formed network ranked according to global efficiency minus cost over cost (Dimitriadis, Salis, et al. 2017). This is repeated for a range of sequentially derived OMSTs, while connections are aggregated to the main network and the objective function *J* computed. The network that maximizes the GE – Cost over Cost value is considered optimal and used as the final reduced network.

$$J_{GCE}^{OMSTs}=GE-Cost$$

An example of the use of OMST can be seen in Supplementary Figure 4, where the original unreduced network is presented next to the reduced network. OMST was utilized for graph reduction purposes in the present analysis, providing the desired sparsity in individual subject’s FCGs. Statistical filtering through surrogate time series has also been implemented prior to OMST (Dimitriadis, Salis, et al. 2017). However, preliminary analyses on the current rs-fMRI dataset using this method resulted in excessively sparse networks with insufficient connections to assess, and interpret, potential group differences. Finally, it must be noted that as a standalone graph reduction / network extraction technique, OMST significantly prevails over surrogate-based methods in terms of speed, an important point especially in studies where MI is used, as its computation is very time consuming.


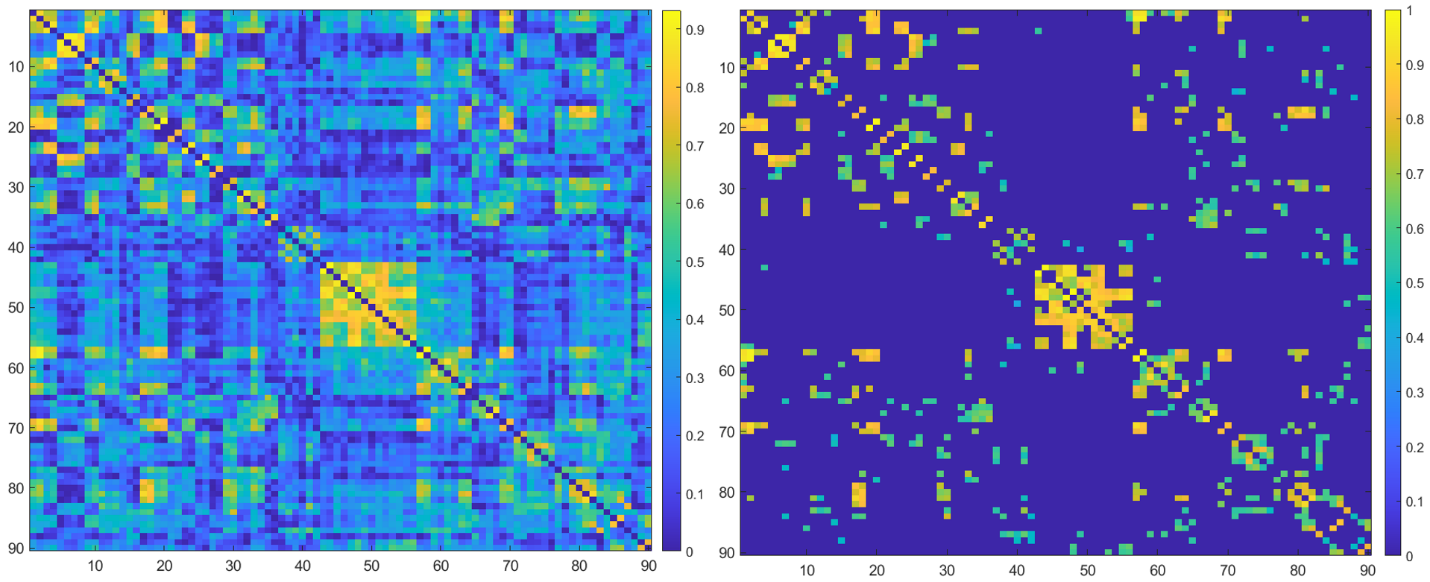


Supplementary figure 4 – Left: FCG based on Pearson correlations, Right: Same FCG after OMST reduction

## Functional Connectivity Estimation

### Static functional connectivity (SFC)

As is typical in Mutual Information (MI) connectivity studies, all edges were divided by the maximum of the matrix to normalize values between 0 and 1. MI-derived FCGs are strictly positive due to the nature of the metric, whereas for the more commonly used Pearson correlation coefficient (Prsn) only positive connections were retained while negative edges were set two zero, as is common practice in network neuroscience studies (Huang et al. 2021; Long et al. 2019; Pedersen et al. 2018; Yang et al. 2020). The interpretation of negative connections is not as straightforward neurobiologically and was not in the scope of the present study.

### Dynamic functional connectivity (DFC)

Several approaches exist for exploring the dynamic functional connectome, in the present work, we opted for one with a relatively fixed goal in terms of network topologies. While in keeping with the data-driven theme, this methodology may potentially lead to more generalizable results than some alternatives that identify network topologies more freely from the data, potentially being more sample-dependent. In the present work cartographic profiling was used to characterize brain states over time. The tapered maximum overlapping sliding window approach utilized in the present study is illustrated in Supplementary Figure 5.


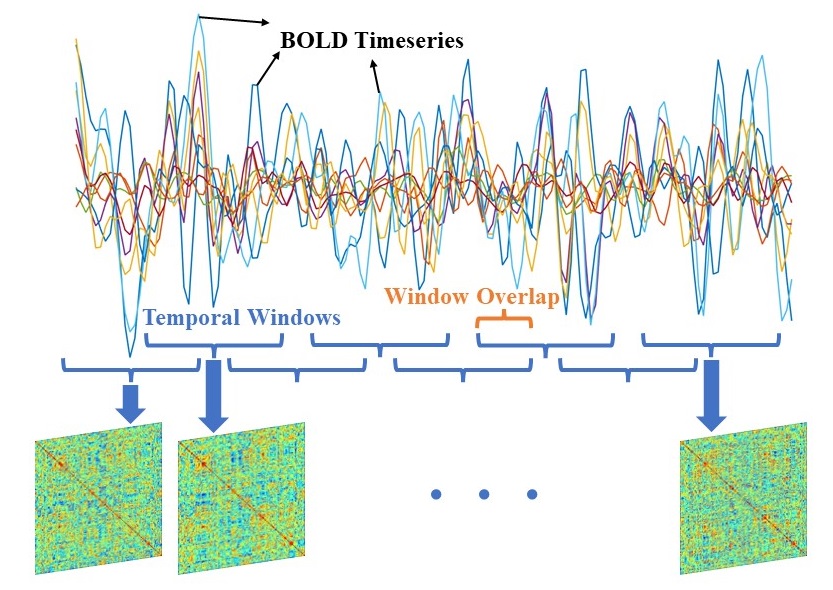


*Supplementary figure 5 Overlapping sliding window for dynamic functional connectivity*

At the core of the DFC analysis employed in the present work, is a brain state identification process based on ”cartographic profiling”, which is illustrated in Supplementary Figure 6. This process characterizes time-resolved networks as predominately integrated or segregated.


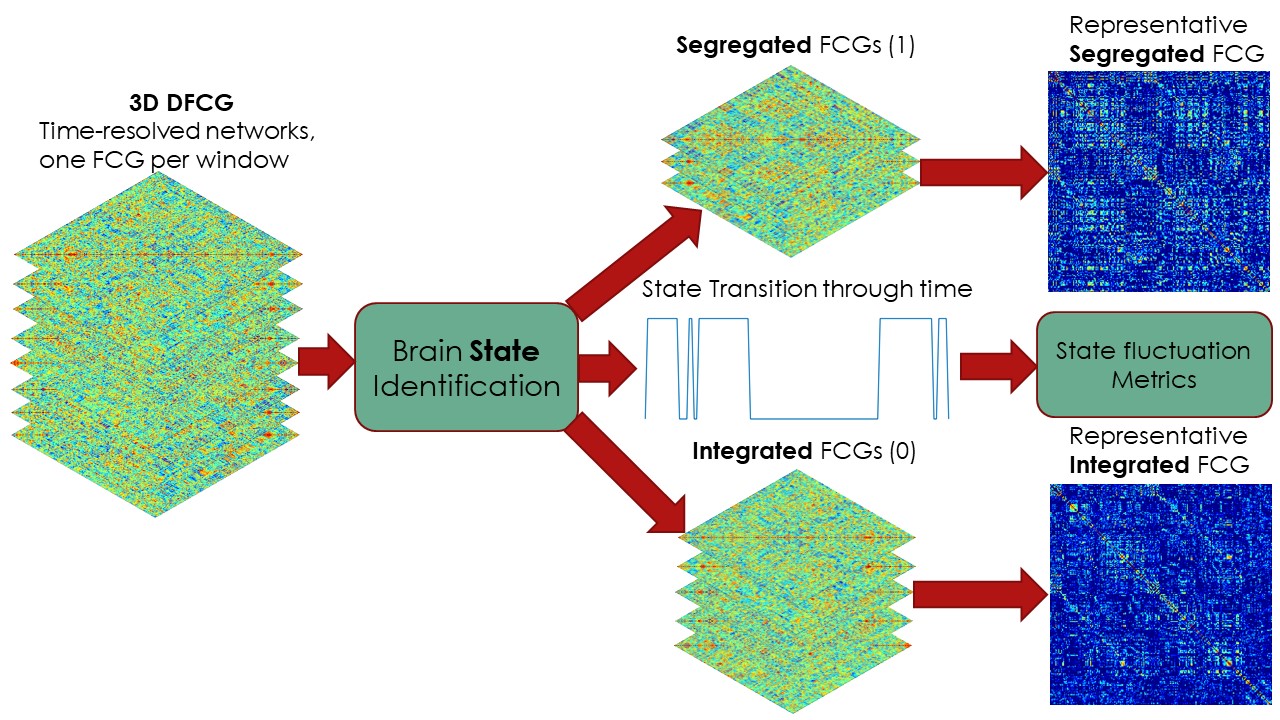


Supplementary figure 6 overview of brain state identification process

The cartographic profile approach was utilized in order to derive the predominant states present in the data and their temporal fluctuation information. State characterization relates to each time-resolved network/DFCG, i.e., the network computed for each temporal window. The first step in this process is the application of the Louvain algorithm described above. Subsequently, modularity assignments were used to calculate two nodal network measures, participation coefficient and within-module degree z-score, also analyzed above. The combination of these metrics was achieved in the form of a joint histogram or “cartographic profile” (Shine et al. 2016). The joint histograms of the two descriminative network measures (for each time-resolved network) were subsequently vectorized and used as feature input to the *k*-means algorithm which was then employed to classify each DFCG, into one of *k*=2 groups. Although the main notion of this procedure requires 2 final states (integrated-segregated), in order to be sure of the actual prevelance of these two main network tendencies in the subject’s data (time-resolved networks), a wide range of clusters were evaluated, *k*=2-7. As can be seen in Supplemetary Figure 7, subject and group-averaged silhouette values (a clustering quality index), suggest that *k*=2 clusters are clearly optimal for the present dataset.


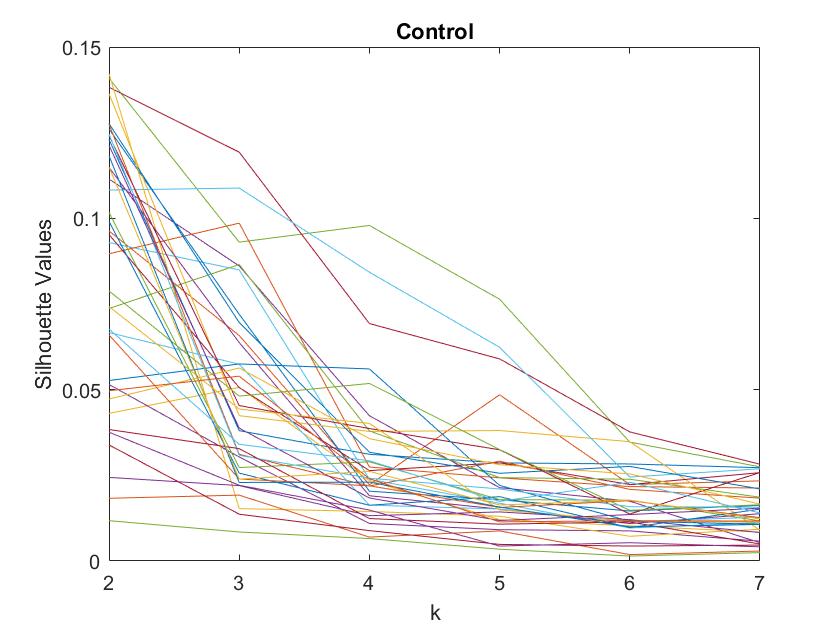

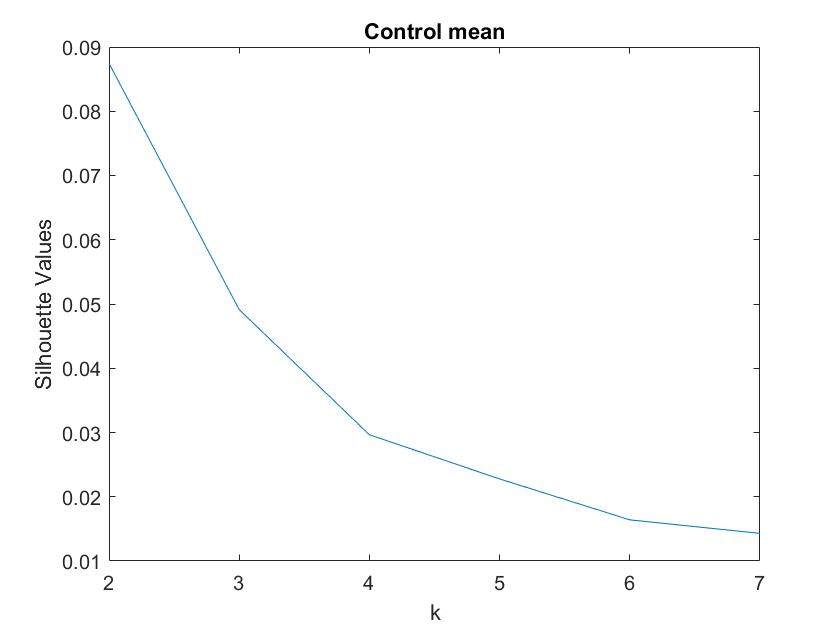

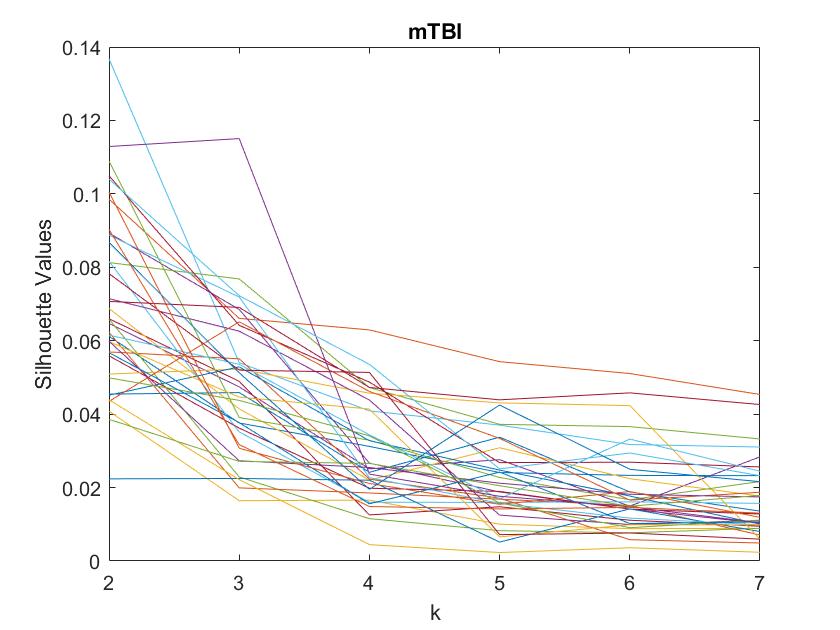

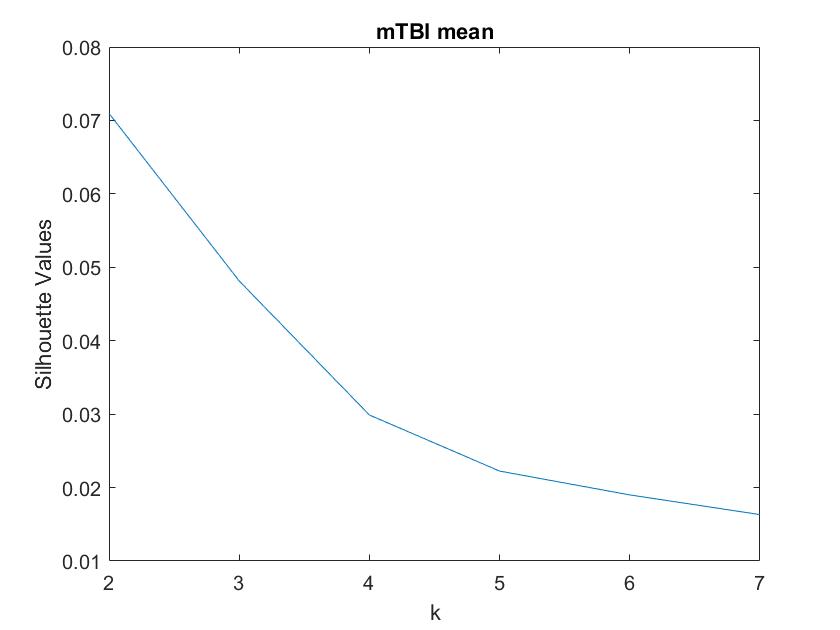


Supplementary figure 7 – Silhouette values for clusters *k*=2-7 for *k*-means
left: individual subject values for mTBI and control groups
right: average silhouette values for each group

Correlation distance was used as the *k*-means distance metric for forming clusters according to previous work (Fukushima et al. 2018; Luppi et al. 2019; Luppi, Carhart-Harris, et al. 2021; Shine et al. 2016). In our own experiments, other well known distance metrics such as squared euclidean, cityblock, cosine and hamming were also tested but did not provide more reliable or significantly different results in terms of computed state representative FCGs or metrics. Multiple re-initializations of the *k*-means clustering algorithm (centroid reinitialization) is recommended by previous studies (500 times) and implemented in the current analysis (through 2000 random restarts) in order to reduce the probability of the outputs being driven by unpredictable initial conditions. From the two identified clusters the one with the highest mean participation coefficient represents the *integrated state*, whereas the second cluster is deemed to represent the *segregated state*. The temporal fluctuation of the classified states constitutes a binary timeseries. The next step after state temporal identification entails the calculation of centroid-representative networks for the integrated and segregated states, computed as the edgewise median of each state’s DFCGs for each subject. In Supplementary Figure 8, an example of a random subject’s predominately integrated and segregated representative FCGs/networks are presented. Upon visual inspection of these matrices/networks one can observe the more segregated network tendencies of the graph on the right compared to the left.


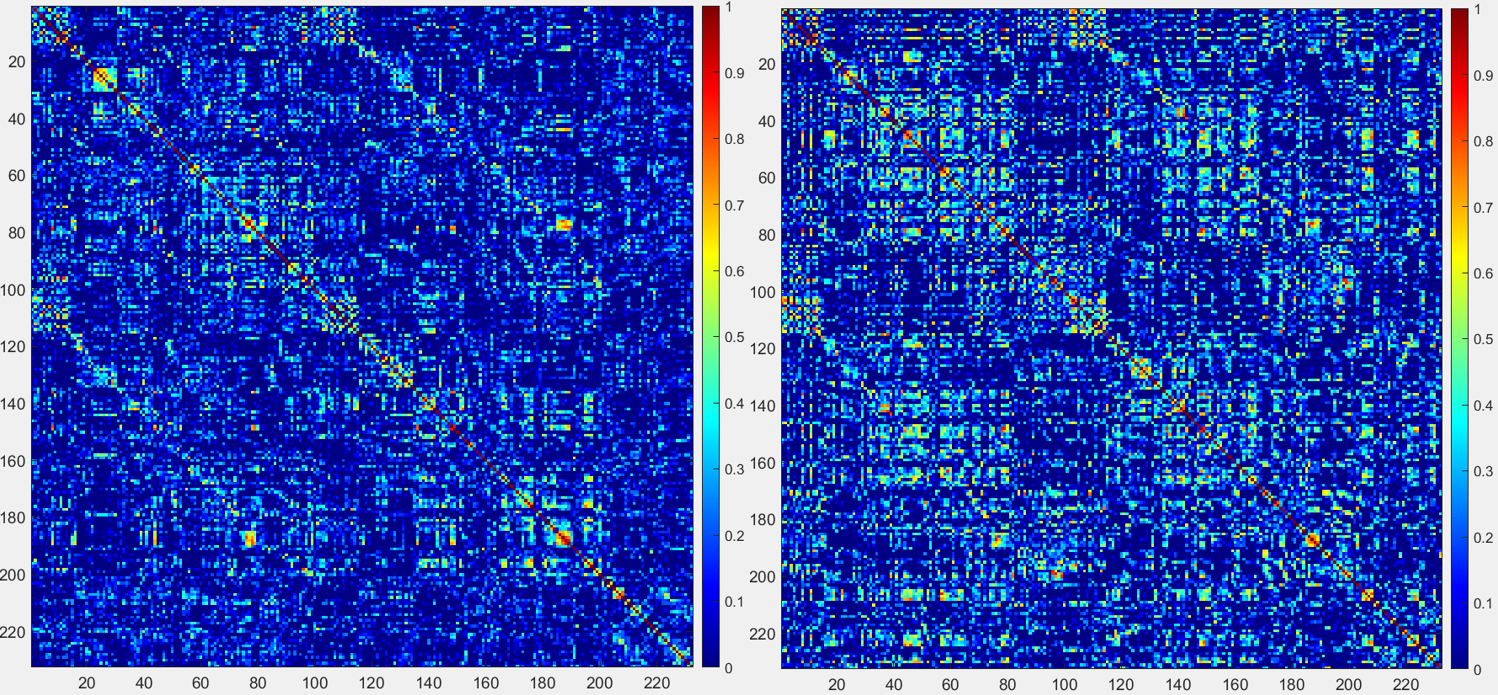


Supplementary figure 8 – predominately integrated FCG (left), segregated (right)

## Machine Learning

The XGBoost classification model was utilized for all five internal estimators of the decision-level fusion machine learning model as well as for the calculation of feature importance rankings in the nested CV feature selection process. XGBoost is an ensemble-type model producing final models similar in structure and operation to Random Forests but with an improved training scheme based on gradient boosting and regularization. For feature selection purposes, the recommended feature importance metric of “gain” was selected as a relatively simple and robust measure, similar to the impurity-based (using the gini metric) feature importance produced by Random Forest models. Very similar model performance and results in terms of selected features were obtained with the Random Forest ensemble classification model. XGBoost was selected mostly due to implementation optimizations that allow for parallel execution and thus significantly improved model training speeds.

## Software and code

MATLAB version 9.8 (R2020a) and Python version 3.6.8 were used for the various analysis parts. Main preprocessing steps were completed in the MATLAB-based toolbox SPM12 (fil.ion.ucl.ac.uk/spm/) and denoising steps were carried out in the SPM-based toolbox CONN (web.conn-toolbox.org/). BrainNet Viewer (Xia, Wang, and He 2013) was used for brain region and connection visualization purposes (freely distributed in nitrc.org/projects/bnv/). Connectivity-related computations were performed using custom MATLAB scripts. The BCT or *Brain Connectivity Toolbox* (sites.google.com/site/bctnet/;(Rubinov and Sporns 2010) was employed to calculate most graph measures. An implementation of the Small-World Propensity measure (Muldoon, Bridgeford, and Bassett 2016) can be found in (complexsystemsupenn.com). Code for topological network filtering with OMST (and several other algorithms) can be found in Dr. Stavros Dimitriadi’s GitHub repository (github.com/stdimitr). Included ML experiments were set up using the python programming language. A python implementation of XGBoost can be found in (xgboost.readthedocs.io/en/latest/python/index.html). Several other ML or data transformation-related operations utilized the NumPy (numpy.org) and sklearn (scikit-learn.org) libraries (as well as pandas and SciPy).

**Supplementary Table 1.** Individual demographic and clinical data, conventional MRI and neuropsychiatric manifestations in mTBI patients.

| **ID** | **Age (years)** | **Edu** | **GCS** | **TPI** | **Lesion** | **Depre-ssion** | **Anxiety** | **Episodic Memory** | **Executive/ Attention** |
| --- | --- | --- | --- | --- | --- | --- | --- | --- | --- |
| 1 | 47 | 12 | 13 | 18 | C (L/RT, L/RF), DAI (RF) | -- | -- | -- | -- |
| 2 | 43 | 6 | 15 | 6 | -- | -- | -- | -- | -- |
| 3 | 45 | 6 | 13 | 5 | C (L/RT, LF) | yes | -- | -- | yes |
| 4 | 19 | 12 | 15 | 36 | -- | -- | -- | -- | yes |
| 5 | 50 | 12 | 15 | 11 | C (L/RT, L/RF) | -- | yes | -- | -- |
| 6 | 64 | 6 | 14 | 15 | C (RT, L/RF) | -- | -- | yes | yes |
| 7 | 63 | 6 | 15 | 30 | DAI (L/RF) | -- | -- | -- | -- |
| 8 | 48 | 7 | 14 | 19 | -- | -- | yes | -- | -- |
| 9 | 64 | 17 | 15 | 12 | -- | -- | yes | yes | -- |
| 10 | 39 | 12 | 14 | 10 | C (RF), DAI (RF) | -- | -- | -- | -- |
| 11 | 50 | 13 | 15 | 20 | C (RF) | -- | -- | yes | yes |
| 12 | 41 | 6 | 15 | 31 | -- | -- | -- | -- | -- |
| 13 | 24 | 12 | 14 | 29 | DAI (L/RF) | -- | -- | -- | -- |
| 14 | 21 | 15 | 14 | 14 | DAI (RF) | -- | yes | -- | yes |
| 15 | 59 | 12 | 15 | 36 | -- | -- | yes | -- | -- |
| 16 | 47 | 8 | 15 | 24 | -- | -- | -- | yes | -- |
| 17 | 66 | 16 | 15 | 15 | -- | yes | yes | -- | -- |
| 18 | 46 | 22 | 15 | 16 | -- | -- | yes | -- | yes |
| 19 | 68 | 6 | 15 | 15 | DAI (L/RF) | yes | yes | -- | -- |
| 20 | 19 | 12 | 15 | 14 | -- | -- | yes | -- | -- |
| 21 | 22 | 12 | 15 | 12 | -- | -- | -- | -- | yes |
| 22 | 30 | 14 | 14 | 60 | C (LT, LF) | -- | -- | -- | yes |
| 23 | 22 | 16 | 15 | 12 | C (LT) | -- | -- | -- | -- |
| 24 | 18 | 12 | 15 | 11 | C (L/RF) | -- | yes | -- | -- |
| 25 | 64 | 12 | 15 | 36 | -- | -- | yes | -- | -- |
| 26 | 36 | 6 | 15 | 16 | C (RF) | yes | yes | -- | -- |
| 27 | 64 | 12 | 15 | 16 | C (RT) | yes | yes | yes | yes |
| 28 | 21 | 15 | 14 | 13 | C (LT), DAI (LT, L/RF) | -- | -- | -- | yes |
| 29 | 19 | 12 | 15 | 36 | -- | -- | -- | yes | yes |
| 30 | 56 | 14 | 15 | 12 | -- | -- | yes | -- | -- |
| 31 | 66 | 17 | 15 | 29 | -- | yes | yes | -- | -- |
| 32 | 18 | 12 | 14 | 6 | C (RT, L/RF), DAI (LT, L/RF) | -- | -- | yes | -- |
| 33 | 45 | 11 | 14 | 6 | C (L/RT) | yes | yes | -- | -- |
| 34 | 28 | 15 | 15 | 11 | DAI (LT, L/RF) | -- | -- | yes | -- |
| 35 | 47 | 12 | 15 | 8 | -- | -- | yes | -- | -- |
| 36 | 52 | 12 | 15 | 14 | -- | yes | yes | -- | -- |
| 37 | 18 | 12 | 13 | 7 | DAI (L/RT) | -- | -- | yes | yes |

Abbreviations; L: Left and R: Right hemisphere, T: Temporal, F: Frontal, GCS: Glasgow Coma Scale, DAI: 1-3 chronic hemorrhagic foci resulting from diffuse axonal injuries, C: gliotic areas due to contusions<=3cm, Edu: Education in years, TPI: Time post injury in months.

# Supplementary References

Antonakakis, Marios et al. 2016. “Altered Cross-Frequency Coupling in Resting-State MEG after Mild Traumatic Brain Injury.” *International Journal of Psychophysiology* 102: 1–11. http://dx.doi.org/10.1016/j.ijpsycho.2016.02.002.

Barttfelda, Pablo et al. 2015. “Signature of Consciousness in the Dynamics of Resting-State Brain Activity.” *Proceedings of the National Academy of Sciences of the United States of America* 112(3): 887–92.

Bassett, Danielle S., and Edward T. Bullmore. 2017. “Small-World Brain Networks Revisited.” *Neuroscientist* 23(5): 499–516.

Dimitriadis, Stavros I., Marios Antonakakis, et al. 2017. “Data-Driven Topological Filtering Based on Orthogonal Minimal Spanning Trees: Application to Multigroup Magnetoencephalography Resting-State Connectivity.” *Brain Connectivity* 7(10): 661–70.

Dimitriadis, Stavros I., Bethany Routley, David E. Linden, and Krish D. Singh. 2018. “Reliability of Static and Dynamic Network Metrics in the Resting-State: A MEG-Beamformed Connectivity Analysis.” *Frontiers in Neuroscience* 12(AUG).

Dimitriadis, Stavros I., Christos Salis, Ioannis Tarnanas, and David E. Linden. 2017. “Topological Filtering of Dynamic Functional Brain Networks Unfolds Informative Chronnectomics: A Novel Data-Driven Thresholding Scheme Based on Orthogonal Minimal Spanning Trees (OMSTs).” *Frontiers in Neuroinformatics* 11(April).

Fukushima, Makoto et al. 2018. “Structure–Function Relationships during Segregated and Integrated Network States of Human Brain Functional Connectivity.” *Brain Structure and Function* 223(3): 1091–1106.

Huang, Danqing et al. 2021. “Childhood Trauma Is Linked to Decreased Temporal Stability of Functional Brain Networks in Young Adults.” *Journal of Affective Disorders* 290(May): 23–30. https://doi.org/10.1016/j.jad.2021.04.061.

Long, Yicheng et al. 2019. “Psychological Resilience Negatively Correlates with Resting-State Brain Network Flexibility in Young Healthy Adults: A Dynamic Functional Magnetic Resonance Imaging Study.” *Annals of Translational Medicine* 7(24): 809–809.

Luppi, Andrea I. et al. 2019. “Consciousness-Specific Dynamic Interactions of Brain Integration and Functional Diversity.” *Nature Communications* 10(1). http://dx.doi.org/10.1038/s41467-019-12658-9.

Luppi, Andrea I., Robin L. Carhart-Harris, et al. 2021. “LSD Alters Dynamic Integration and Segregation in the Human Brain.” *NeuroImage* 227(November 2020).

Luppi, Andrea I, Helena M Gellersen, et al. 2021. “Searching for Consistent Brain Network Topologies Across the Garden of (Shortest) Forking Paths.” *bioRxiv*: 2021.07.13.452257. https://www.biorxiv.org/content/10.1101/2021.07.13.452257v1%0Ahttps://www.biorxiv.org/content/10.1101/2021.07.13.452257v1.abstract.

Muldoon, Sarah Feldt, Eric W. Bridgeford, and Danielle S. Bassett. 2016. “Small-World Propensity and Weighted Brain Networks.” *Scientific Reports* 6(February): 1–13. http://dx.doi.org/10.1038/srep22057.

Newman, M. E.J. 2004. “Fast Algorithm for Detecting Community Structure in Networks.” *Physical Review E - Statistical Physics, Plasmas, Fluids, and Related Interdisciplinary Topics* 69(6): 5.

Pedersen, Mangor, Andrew Zalesky, Amir Omidvarnia, and Graeme D. Jackson. 2018. “Multilayer Network Switching Rate Predicts Brain Performance.” *Proceedings of the National Academy of Sciences of the United States of America* 115(52): 13376–81.

Rubinov, Mikail, and Olaf Sporns. 2010. “Complex Network Measures of Brain Connectivity: Uses and Interpretations.” *NeuroImage* 52(3): 1059–69. http://dx.doi.org/10.1016/j.neuroimage.2009.10.003.

———. 2011. “Weight-Conserving Characterization of Complex Functional Brain Networks.” *NeuroImage* 56(4): 2068–79.

Saba, Valentina et al. 2019. “Brain Connectivity and Information-Flow Breakdown Revealed by a Minimum Spanning Tree-Based Analysis of Mri Data in Behavioral Variant Frontotemporal Dementia.” *Frontiers in Neuroscience* 13(March): 1–16.

Shine, James M. et al. 2016. “The Dynamics of Functional Brain Networks: Integrated Network States during Cognitive Task Performance.” *Neuron* 92(2): 544–54. http://dx.doi.org/10.1016/j.neuron.2016.09.018.

Watts, Duncan J., and Steven H. Strogatz. 1998. “Collective Dynamics of ‘Small-World’ Networks.” *NATURE* 393: 440–42.

Xia, Mingrui, Jinhui Wang, and Yong He. 2013. “BrainNet Viewer: A Network Visualization Tool for Human Brain Connectomics.” *PLoS ONE* 8(7).

Yang, Jie et al. 2020. “Connectomic Underpinnings of Working Memory Deficits in Schizophrenia: Evidence from a Replication FMRI Study.” *Schizophrenia Bulletin* 46(4): 916–26.
